# Supplementary material for: Nitrogen starvation reveals the mitotic potential of mutants in the S/MAPK pathways
Source: Nat Commun. 2020 Apr 24;11:1973. doi: 10.1038/s41467-020-15880-y (PMC7181643; doi:10.1038/s41467-020-15880-y)
Supplement: Supplementary file 1 — Supplementary Information [file 41467_2020_15880_MOESM1_ESM.pdf]

Nitrogen starvation Reveals the Mitotic Potential of Mutants in the S/MAPK Pathways

Rostyslav Makarenko[1,2¶], Claire Denis[1¶], Stefania Francesconi[1¶], Serge Gangloff[1], and Benoît Arcangioli[1*]

[1] Genome Dynamics Unit, Institut Pasteur, UMR3525 CNRS, Paris 75015, France

[2] Sorbonne Université, École Doctorale 515, F-75005 Paris, France

* Corresponding author

E-mail: benoit.arcangioli@pasteur.fr

Running Tittle: S/MAPK mutants increase the fitness of quiescence

Key Words: Quiescence, Mutations, S/MAPK, RpoS, Adaptation, Kin selection

¶These authors contributed equally to this work

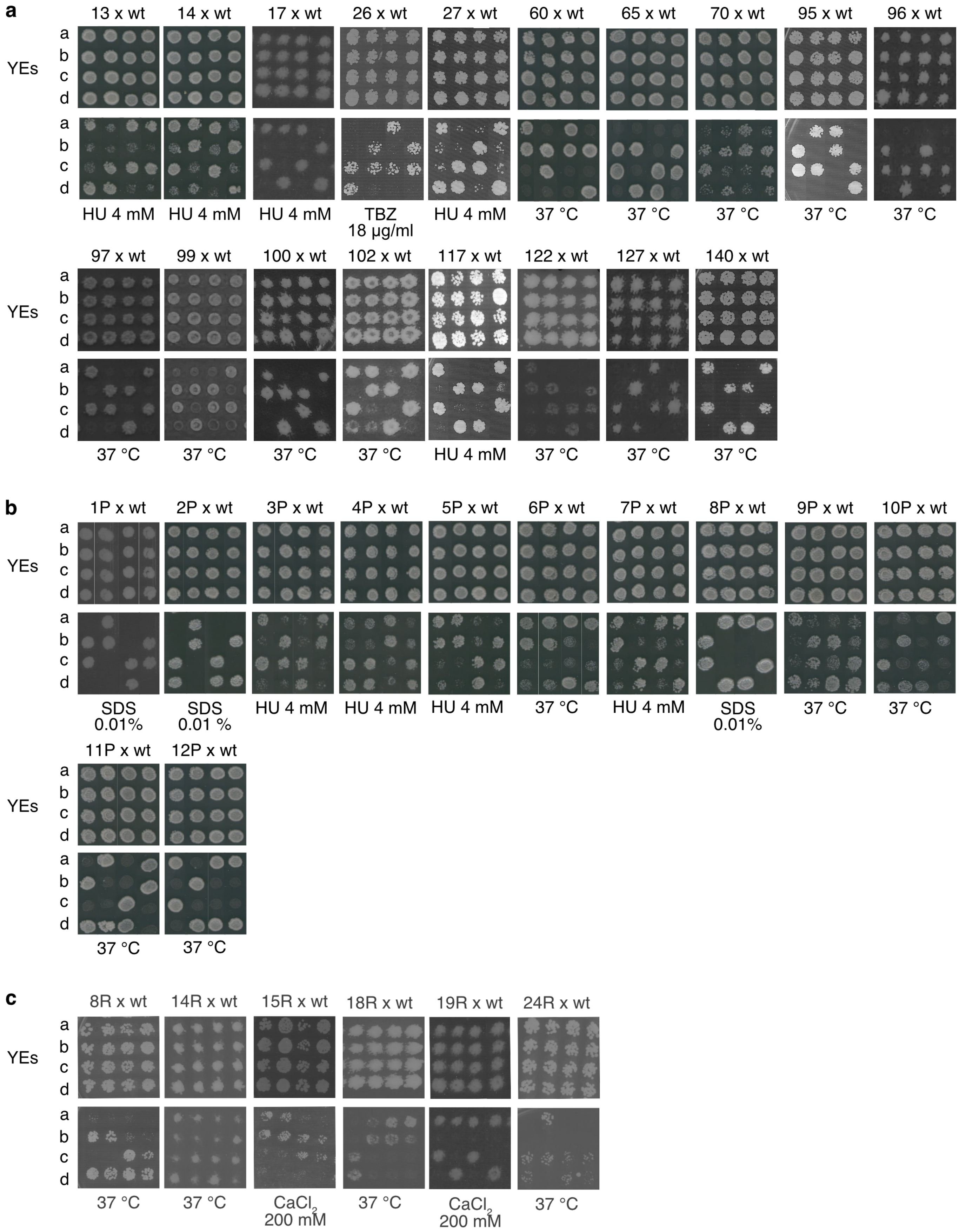

Supplementary Figure 1. Mendelian segregation of the mutations causing a sensitivity phenotype. The mutants from the 30 initially identified clones and the 6 identified later in the 36 randomly picked clones (Supplementary Table 3) were crossed with a PΔ17 prototrophic strain, sporulated and dissected. Replica plating or spotting was performed on the media containing the various drugs at the given concentration. Four representative tetrads are shown for a sensitivity phenotype for each strain.

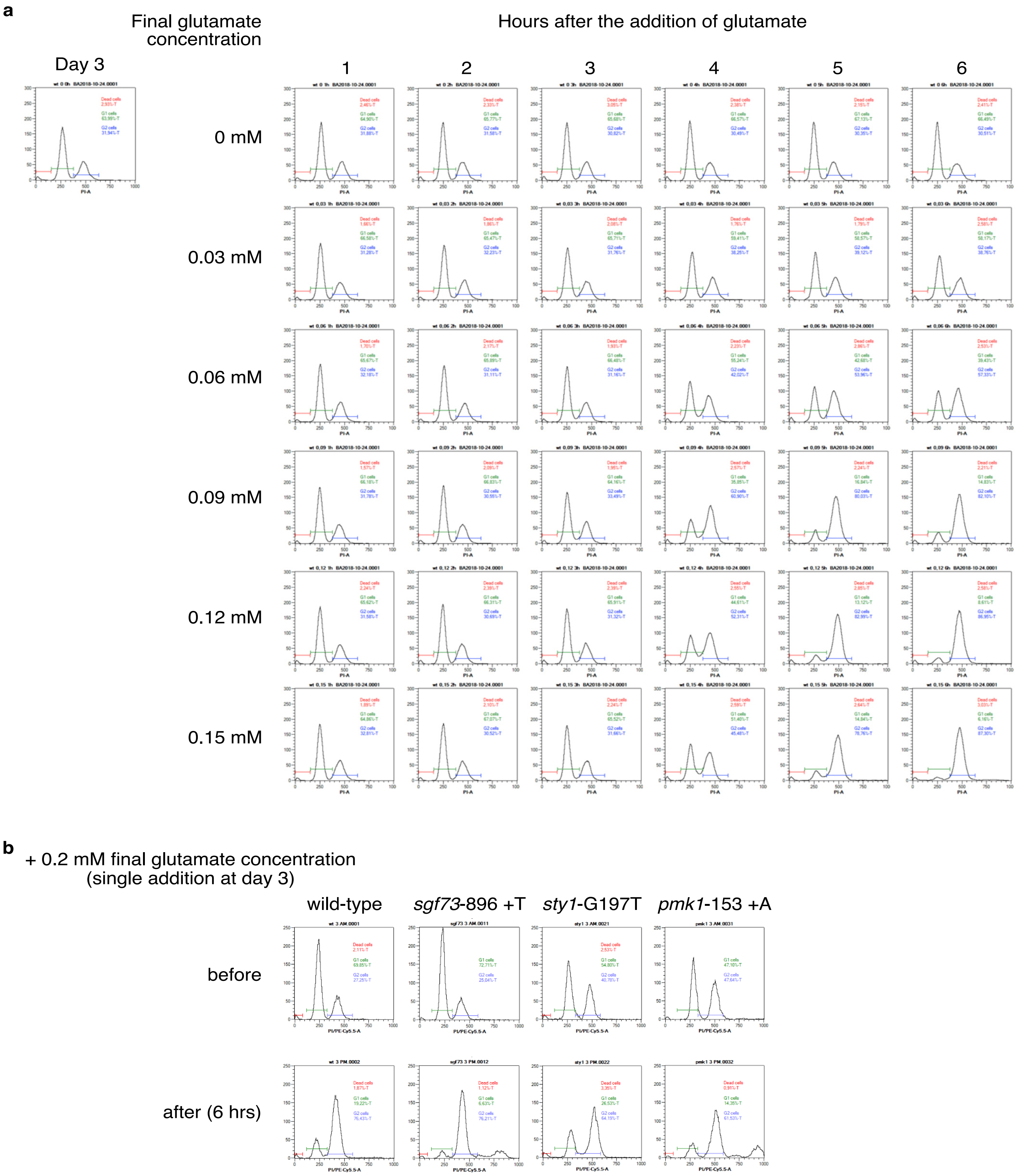

Supplementary Figure 2. Determination of the amount of nitrogen necessary to trigger a physiological response. (a) Effect of glutamate concentration on the cell cycle of a three-day-old quiescent culture of a wild-type strain. FACS analysis of the DNA content was followed for six hours after the addition of glutamate at the indicated final concentration. (b) Effect of glutamate addition at a final concentration of 0.2 mM on the cell cycle of three-day--old quiescent cultures of S/MAPK mutants. FACS analysis of the DNA content of strains determined before and six hours after the addition.

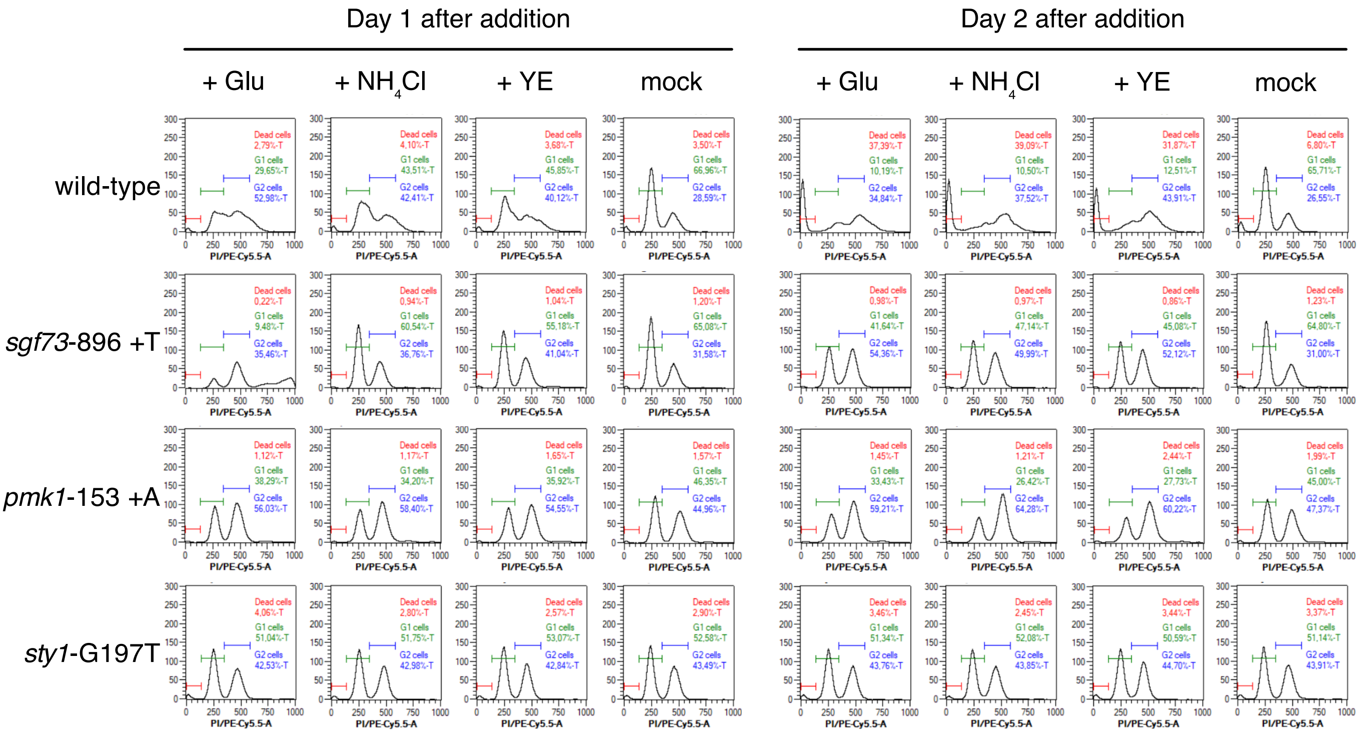

Supplementary Figure 3. DNA content at days 1 and 2 after the first addition. This figure extends Figure 6. Effect of the addition of various nitrogen sources to three-day-old quiescent cultures (day 0) twice a day (mornings and evenings) determined by FACS analysis in the morning. Glutamate (5g/L), ammonium chloride (1.6 g/L) or yeast extract (5 g/L) was added at 1/1,500 of the volume of the culture at each addition. DNA content analysis of 10,000 ethanol fixed cells was determined by FACS.

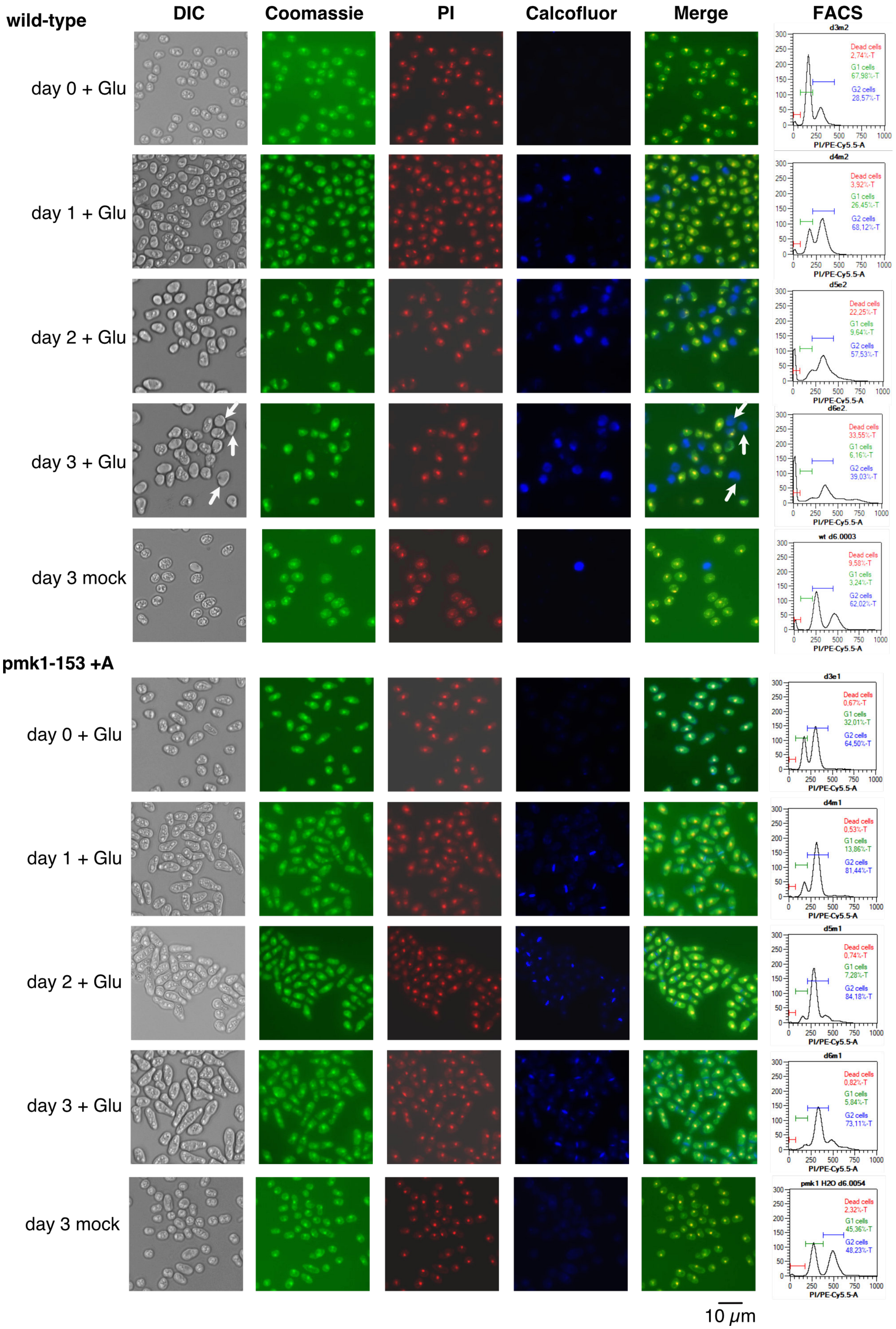
wild-type
DIC
Coomassie
PI
Calcofluor
Merge
FACS
day 0 + Glu
day 1 + Glu
day 2 + Glu
day 3 + Glu
day 3 mock
d3m2
Dead cells 2,74%-T
G1 cells 67,98%-T
G2 cells 28,57%-T
d4m2
Dead cells 3,92%-T
G1 cells 26,45%-T
G2 cells 68,12%-T
d5e2
Dead cells 22,25%-T
G1 cells 9,64%-T
G2 cells 57,53%-T
d6e2
Dead cells 33,55%-T
G1 cells 6,16%-T
G2 cells 39,03%-T
wt d6.0003
Dead cells 9,58%-T
G1 cells 3,24%-T
G2 cells 62,02%-T
PI/PE-Cy5.5-A
pmk1-153 +A
day 0 + Glu
day 1 + Glu
day 2 + Glu
day 3 + Glu
day 3 mock
d3e1
Dead cells 0,67%-T
G1 cells 32,01%-T
G2 cells 64,50%-T
d4m1
Dead cells 0,53%-T
G1 cells 13,86%-T
G2 cells 81,44%-T
d5m1
Dead cells 0,74%-T
G1 cells 7,28%-T
G2 cells 84,18%-T
d6m1
Dead cells 0,82%-T
G1 cells 5,84%-T
G2 cells 73,11%-T
pmk1 H2O d6.0054
Dead cells 2,32%-T
G1 cells 45,36%-T
G2 cells 48,23%-T
PI/PE-Cy5.5-A
10 μm

Supplementary Figure 4. Cellular response to the repeated addition of traces of glutamate to three-day-old quiescent cultures. Day 0 is before the first addition. Cells at the indicated time points were heat fixed at 60 °C on a glass slide and stained with a solution containing equal volumes of 0.05% Coomassie Brilliant Blue G in PBS (proteins staining), 0.1 µg/mL of propidium iodide (PI) (nucleic acids staining) and 25 µg/mL of Calcofluor white (staining of the septum in cells that have passed mitosis). Images were taken using a Zeiss Axioplan 2 Imaging M epifluorescence microscope equipped with an AxioCam MRm digital camera and Zen 2 software. When provided with traces of glutamate, only the wild-type culture accumulates cells with a smooth appearance under differential-interference-contrast (DIC) microscopy that stain brightly with Calcofluor. These cells are negative for both Coomassie and propidium iodide staining as shown in the merged images. White arrows point to some of these cells. The accumulation of this population correlates with the fraction of propidium negative cells observed by FACS analysis. Unlike wild-type, all the *pmk1*-153 +A mutant cells are positive for the three dyes and Calcofluor specifically stains the septum of binucleated cells that have passed mitosis. The presented micrographs are representative of the situation observed in the field of 20 pictures each from 2 independent experiments.

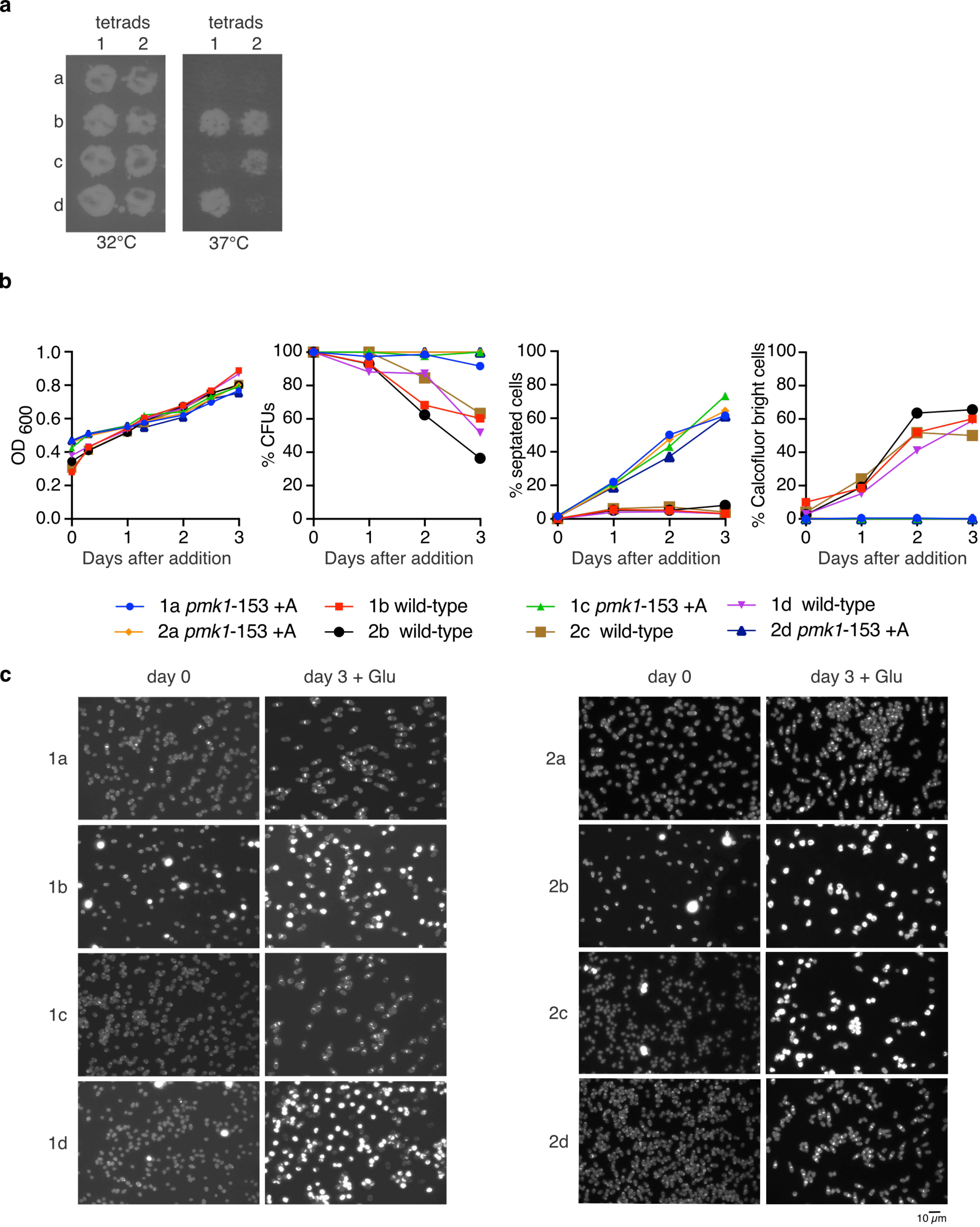

Supplementary Figure 5. Scavenging behavior of the *pmk1* mutant crossed into the 972 background. The PΔ17 *pmk1*-153 +A mutant was crossed with the 972 h^- strain and two full tetrads were analyzed after supplementing traces of glutamate to three-day-old cultures (day 0) as described in Figure 6. (a) The thermosensitive phenotype conferred by the *pmk1*-153 +A allele segregates 2:2 in the spores of both tetrads used in this experiment. (b) Cell mass increase (OD_{600nm}), colony forming units, % of septated cells and % of Calcofluor white bright cells were determined for 3 days following the first addition of glutamate. A single determination for each spore is presented (c) Images taken with a Zeiss Axioplan 2 Imaging M epifluorescence microscope on days 0 and 3 after the first supplement of glutamate of fixed cells stained with Calcofluor white (25 µg/mL) that highlights septum formation in cells that have passed mitosis or cells devoid of nucleic acids and proteins. The presented micrographs are representative of the situation observed in the field of 20 pictures from each spore.

Supplementary Table 1: Differences between PB1623 and 972 h- established on 243 sequenced genomes - Publicly available at the NCBI BioProject database (accession no: PRJNA413662)

<table>
<tr><th colspan="7">SNVs</th></tr>
<tr><th>#CHROM</th><th>POS</th><th>ID</th><th>REF</th><th>ALT</th><th>QUAL</th><th></th></tr>
<tr><td>II</td><td>731642</td><td>.</td><td>G</td><td>T</td><td>3548.0</td><td>EFF=NON_SYNONYMOUS_CODING(MODERATE|MISSENSE|Gat/Tat|D110Y||SPBC119.08.1:exon:1|||transcript:SPBC119.08.1|4|1)</td></tr>
<tr><td>I</td><td>5115257</td><td>.</td><td>C</td><td>G</td><td>2997.0</td><td>EFF=NON_SYNONYMOUS_CODING(MODERATE|MISSENSE|gaG/gaC|E386D||SPAC29A4.16.1:exon:1|||transcript:SPAC29A4.16.1|1|1)</td></tr>
<tr><td>II</td><td>1048712</td><td>.</td><td>G</td><td>A</td><td>2737.0</td><td>EFF=NON_SYNONYMOUS_CODING(MODERATE|MISSENSE|gCc/gTc|A240V||SPBC337.10c.1:exon:1|||transcript:SPBC337.10c.1|1|1),SYNONYMOUS_CODING(LOW|SILENT|cgG/cgA|R96||SPNCRNA.1407.1:exon:1|||transcript:SPNCRNA.1407.1|1|1|WARNING_TRANSCRIPT_MULTIPLE_STOP_CODONS)</td></tr>
<tr><td>II</td><td>1238931</td><td>.</td><td>G</td><td>A</td><td>2532.0</td><td>EFF=INTRON(MODIFIER|||||SPBC651.01c.1:exon:1|||transcript:SPBC651.01c.1|2|1)</td></tr>
<tr><td>II</td><td>1987098</td><td>.</td><td>C</td><td>A</td><td>2502.0</td><td>EFF=SPLICE_SITE_REGION(LOW|||||SPBC1A4.06c.1:exon:1|||transcript:SPBC1A4.06c.1|4|1),SYNONYMOUS_CODING(LOW|SILENT|ctG/ctT|L376||SPBC1A4.06c.1:exon:1|||transcript:SPBC1A4.06c.1|4|1),UTR_3_PRIME(MODIFIER||427|||SPBC1A4.05.1:exon:1|||transcript:SPBC1A4.05.1|2|1)</td></tr>
<tr><td>I</td><td>477739</td><td>.</td><td>T</td><td>C</td><td>2305.0</td><td>EFF=NON_SYNONYMOUS_CODING(MODERATE|MISSENSE|Att/Gtt|I304V||SPAC24H6.06.1:exon:1|||transcript:SPAC24H6.06.1|2|1)</td></tr>
<tr><td>II</td><td>879033</td><td>.</td><td>T</td><td>A</td><td>2303.0</td><td>EFF=SYNONYMOUS_CODING(LOW|SILENT|gtT/gtA|V510||SPBC713.06.1:exon:1|||transcript:SPBC713.06.1|1|1),UTR_3_PRIME(MODIFIER||886|||SPBC713.07c.1:exon:1|||transcript:SPBC713.07c.1|1|1)</td></tr>
<tr><td>I</td><td>1999192</td><td>.</td><td>G</td><td>C</td><td>2296.0</td><td>EFF=NON_SYNONYMOUS_CODING(MODERATE|MISSENSE|Cgg/Ggg|R34G||SPAC767.01c.1:exon:1|||transcript:SPAC767.01c.1|2|1),NON_SYNONYMOUS_CODING(MODERATE|MISSENSE|Gta/Cta|V20L||SPNCRNA.785.1:exon:1|||transcript:SPNCRNA.785.1|1|1|WARNING_TRANSCRIPT_MULTIPLE_STOP_CODONS)</td></tr>
<tr><td>II</td><td>1849777</td><td>.</td><td>G</td><td>A</td><td>2255.0</td><td>EFF=NON_SYNONYMOUS_CODING(MODERATE|MISSENSE|aCc/aTc|T1496I||SPBC28E12.06c.1:exon:1|||transcript:SPBC28E12.06c.1|1|1),UTR_3_PRIME(MODIFIER||3611|||SPBC28E12.05.1:exon:1|||transcript:SPBC28E12.05.1|1|1)</td></tr>
<tr><td>I</td><td>3074001</td><td>.</td><td>C</td><td>T</td><td>2232.0</td><td>EFF=NON_SYNONYMOUS_CODING(MODERATE|MISSENSE|aCc/aTc|T275I||SPAC24C9.14.1:exon:1|||transcript:SPAC24C9.14.1|1|1)</td></tr>
<tr><td>I</td><td>5115148</td><td>.</td><td>G</td><td>C</td><td>2210.0</td><td>EFF=NON_SYNONYMOUS_CODING(MODERATE|MISSENSE|Ctg/Gtg|L423V||SPAC29A4.16.1:exon:1|||transcript:SPAC29A4.16.1|1|1)</td></tr>
<tr><td>I</td><td>3583106</td><td>.</td><td>G</td><td>A</td><td>2205.0</td><td>EFF=INTERGENIC(MODIFIER||||||||||1)</td></tr>
<tr><td>II</td><td>923543</td><td>.</td><td>C</td><td>A</td><td>2159.0</td><td>EFF=NON_SYNONYMOUS_CODING(MODERATE|MISSENSE|gaC/gaA|D273E||SPBC646.02.1:exon:1|||transcript:SPBC646.02.1|1|1)</td></tr>
<tr><td>I</td><td>527156</td><td>.</td><td>C</td><td>A</td><td>2153.0</td><td>EFF=STOP_GAINED(HIGH|NONSENSE|taC/taA|Y172*||SPNCRNA.659.1:exon:1|||transcript:SPNCRNA.659.1|1|1|WARNING_TRANSCRIPT_INCOMPLETE)</td></tr>
<tr><td>III</td><td>1765401</td><td>.</td><td>G</td><td>T</td><td>2059.0</td><td>EFF=INTERGENIC(MODIFIER||||||||||1)</td></tr>
<tr><td>I</td><td>3119299</td><td>.</td><td>G</td><td>A</td><td>2044.0</td><td>EFF=UTR_3_PRIME(MODIFIER||80|||SPAC688.07c.1:exon:1|||transcript:SPAC688.07c.1|1|1)</td></tr>
<tr><td>I</td><td>3111508</td><td>.</td><td>A</td><td>G</td><td>2034.0</td><td>EFF=NON_SYNONYMOUS_CODING(MODERATE|MISSENSE|gAa/gGa|E375G||SPAC589.12.1:exon:1|||transcript:SPAC589.12.1|2|1)</td></tr>
<tr><td>II</td><td>685728</td><td>.</td><td>G</td><td>A</td><td>2014.0</td><td>EFF=INTERGENIC(MODIFIER||||||||||1)</td></tr>
<tr><td>II</td><td>4122326</td><td>.</td><td>T</td><td>C</td><td>2009.0</td><td>EFF=NON_SYNONYMOUS_CODING(MODERATE|MISSENSE|Tat/Cat|Y478H||SPNCRNA.1671.1:exon:1|||transcript:SPNCRNA.1671.1|1|1|WARNING_TRANSCRIPT_INCOMPLETE),NON_SYNONYMOUS_CODING(MODERATE|MISSENSE|atA/atG|I101M||SPBC56F2.03.1:exon:1|||transcript:SPBC56F2.03.1|1|1)</td></tr>
<tr><td>I</td><td>3179238</td><td>.</td><td>G</td><td>C</td><td>2006.0</td><td>EFF=STOP_LOST(HIGH|MISSENSE|taG/taC|*436Y||SPNCRNA.893.1:exon:1|||transcript:SPNCRNA.893.1|1|1|WARNING_TRANSCRIPT_INCOMPLETE)</td></tr>
<tr><td>I</td><td>2097159</td><td>.</td><td>C</td><td>T</td><td>1946.0</td><td>EFF=SYNONYMOUS_CODING(LOW|SILENT|aaG/aaA|K209||SPAC25A8.03c.1:exon:1|||transcript:SPAC25A8.03c.1|1|1)</td></tr>
<tr><td>II</td><td>4538164</td><td>.</td><td>A</td><td>T</td><td>1945.0</td><td>EFF=INTERGENIC(MODIFIER||||||||||1)</td></tr>
<tr><td>III</td><td>748812</td><td>.</td><td>A</td><td>T</td><td>1929.0</td><td>EFF=NON_SYNONYMOUS_START(LOW|MISSENSE|Atg/Ttg|M1L||SPCC14G10.04.1:exon:1|||transcript:SPCC14G10.04.1|1|1)</td></tr>
<tr><td>III</td><td>2128341</td><td>.</td><td>C</td><td>T</td><td>1911.0</td><td>EFF=NON_SYNONYMOUS_CODING(MODERATE|MISSENSE|gGc/gAc|G370D||SPCC126.07c.1:exon:1|||transcript:SPCC126.07c.1|1|1)</td></tr>
<tr><td>I</td><td>4919603</td><td>.</td><td>A</td><td>G</td><td>1862.0</td><td>EFF=INTERGENIC(MODIFIER||||||||||1)</td></tr>
<tr><td>II</td><td>438465</td><td>.</td><td>G</td><td>A</td><td>1858.0</td><td>EFF=NON_SYNONYMOUS_CODING(MODERATE|MISSENSE|Cgt/Tgt|R105C||SPBC582.10c.1:exon:1|||transcript:SPBC582.10c.1|1|1),NON_SYNONYMOUS_CODING(MODERATE|MISSENSE|cGt/cAt|R207H||SPNCRNA.1357.1:exon:1|||transcript:SPNCRNA.1357.1|1|1|WARNING_TRANSCRIPT_INCOMPLETE)</td></tr>
<tr><td>II</td><td>1422317</td><td>.</td><td>T</td><td>A</td><td>1852.0</td><td>EFF=SYNONYMOUS_CODING(LOW|SILENT|ctA/ctT|L531||SPBC691.02c.1:exon:1|||transcript:SPBC691.02c.1|1|1)</td></tr>
<tr><td>II</td><td>700113</td><td>.</td><td>A</td><td>T</td><td>1848.0</td><td>EFF=NON_SYNONYMOUS_CODING(MODERATE|MISSENSE|ttT/ttA|F565L||SPNCRNA.1381.1:exon:1|||transcript:SPNCRNA.1381.1|1|1|WARNING_TRANSCRIPT_INCOMPLETE)</td></tr>
<tr><td>III</td><td>1374938</td><td>.</td><td>A</td><td>T</td><td>1813.0</td><td>EFF=NON_SYNONYMOUS_CODING(MODERATE|MISSENSE|tAt/tTt|Y256F||SPCC1281.01.1:exon:1|||transcript:SPCC1281.01.1|1|1)</td></tr>
<tr><td>I</td><td>759334</td><td>.</td><td>G</td><td>T</td><td>1786.0</td><td>EFF=NON_SYNONYMOUS_CODING(MODERATE|MISSENSE|aCt/aAt|T406N||SPAC4G8.03c.1:exon:1|||transcript:SPAC4G8.03c.1|1|1)</td></tr>
<tr><td>III</td><td>679772</td><td>.</td><td>T</td><td>C</td><td>1770.0</td><td>EFF=UTR_5_PRIME(MODIFIER||590|||SPCC16C4.09.1:exon:1|||transcript:SPCC16C4.09.1|1|1)</td></tr>
<tr><td>II</td><td>943554</td><td>.</td><td>C</td><td>A</td><td>1749.0</td><td>EFF=NON_SYNONYMOUS_CODING(MODERATE|MISSENSE|aCa/aAa|T130K||SPBC646.11.1:exon:1|||transcript:SPBC646.11.1|4|1),UTR_3_PRIME(MODIFIER||1548|||SPBC646.12c.1:exon:1|||transcript:SPBC646.12c.1|1|1)</td></tr>
<tr><td>I</td><td>862964</td><td>.</td><td>G</td><td>T</td><td>1737.0</td><td>EFF=INTRON(MODIFIER|||||SPAC23G3.02c.1:exon:1|||transcript:SPAC23G3.02c.1|1|1)</td></tr>
<tr><td>II</td><td>1159678</td><td>.</td><td>G</td><td>C</td><td>1726.0</td><td>EFF=NON_SYNONYMOUS_CODING(MODERATE|MISSENSE|Cgc/Ggc|R391G||SPNCRNA.1417.1:exon:1|||transcript:SPNCRNA.1417.1|1|1|WARNING_TRANSCRIPT_INCOMPLETE),NON_SYNONYMOUS_CODING(MODERATE|MISSENSE|cGc/cCc|R362P||SPBC409.11.1:exon:1|||transcript:SPBC409.11.1|1|1)</td></tr>
<tr><td>I</td><td>1783458</td><td>.</td><td>C</td><td>T</td><td>1706.0</td><td>EFF=NON_SYNONYMOUS_CODING(MODERATE|MISSENSE|aGa/aAa|R543K||SPAC17A5.15c.1:exon:1|||transcript:SPAC17A5.15c.1|1|1)</td></tr>
<tr><td>I</td><td>5400080</td><td>.</td><td>C</td><td>T</td><td>1704.0</td><td>EFF=SYNONYMOUS_CODING(LOW|SILENT|caC/caT|H281||SPAC3G6.11.1:exon:1|||transcript:SPAC3G6.11.1|3|1)</td></tr>
<tr><td>I</td><td>4375421</td><td>.</td><td>A</td><td>T</td><td>1697.0</td><td>EFF=UTR_5_PRIME(MODIFIER||78|||SPAC1527.01.1:exon:1|||transcript:SPAC1527.01.1|1|1)</td></tr>
<tr><td>III</td><td>1862803</td><td>.</td><td>A</td><td>G</td><td>1629.0</td><td>EFF=INTERGENIC(MODIFIER||||||||||1)</td></tr>
<tr><td>II</td><td>2228775</td><td>.</td><td>T</td><td>G</td><td>1617.0</td><td>EFF=INTRON(MODIFIER|||||SPBC14C8.14c.1:exon:1|||transcript:SPBC14C8.14c.1|1|1),UTR_3_PRIME(MODIFIER||2632|||SPBC14C8.13.1:exon:1|||transcript:SPBC14C8.13.1|1|1)</td></tr>
<tr><td>II</td><td>1784826</td><td>.</td><td>G</td><td>A</td><td>1611.0</td><td>EFF=NON_SYNONYMOUS_CODING(MODERATE|MISSENSE|aCt/aTt|T392I||SPBC18H10.08c.1:five_prime_UTR:1|||transcript:SPBC18H10.08c.1|4|1),UTR_3_PRIME(MODIFIER||820|||SPBC18H10.07.1:exon:1|||transcript:SPBC18H10.07.1|2|1)</td></tr>
<tr><td>II</td><td>1672859</td><td>.</td><td>C</td><td>T</td><td>1610.0</td><td>EFF=NON_SYNONYMOUS_CODING(MODERATE|MISSENSE|Cgg/Tgg|R760W||SPBC19C2.01.1:exon:1|||transcript:SPBC19C2.01.1|1|1),NON_SYNONYMOUS_CODING(MODERATE|MISSENSE|Gac/Aac|D794N||SPNCRNA.1451.1:exon:1|||transcript:SPNCRNA.1451.1|1|1|WARNING_TRANSCRIPT_MULTIPLE_STOP_CODONS)</td></tr>
<tr><td>I</td><td>523603</td><td>.</td><td>G</td><td>A</td><td>1607.0</td><td>EFF=NON_SYNONYMOUS_CODING(MODERATE|MISSENSE|Gcg/Acg|A120T||SPAC227.15.1:exon:1|||transcript:SPAC227.15.1|1|1)</td></tr>
<tr><td>II</td><td>1962419</td><td>.</td><td>T</td><td>A</td><td>1590.0</td><td>EFF=UTR_5_PRIME(MODIFIER||602|||SPBC1E8.03c.1:exon:1|||transcript:SPBC1E8.03c.1|1|1)</td></tr>
<tr><td>II</td><td>2228777</td><td>.</td><td>G</td><td>T</td><td>1572.0</td><td>EFF=INTRON(MODIFIER|||||SPBC14C8.14c.1:exon:1|||transcript:SPBC14C8.14c.1|1|1),UTR_3_PRIME(MODIFIER||2634|||SPBC14C8.13.1:exon:1|||transcript:SPBC14C8.13.1|1|1)</td></tr>
<tr><td>II</td><td>635763</td><td>.</td><td>G</td><td>A</td><td>1562.0</td><td>EFF=UTR_5_PRIME(MODIFIER||313|||SPBC115.03.1:five_prime_UTR:1|||transcript:SPBC115.03.1|1|1)</td></tr>
<tr><td>II</td><td>1673002</td><td>.</td><td>G</td><td>C</td><td>1558.0</td><td>EFF=NON_SYNONYMOUS_CODING(MODERATE|MISSENSE|aCg/aGg|T746R||SPNCRNA.1451.1:exon:1|||transcript:SPNCRNA.1451.1|1|1|WARNING_TRANSCRIPT_MULTIPLE_STOP_CODONS),SYNONYMOUS_CODING(LOW|SILENT|acG/acC|T807||SPBC19C2.01.1:exon:1|||transcript:SPBC19C2.01.1|1|1)</td></tr>
<tr><td>II</td><td>1962439</td><td>.</td><td>A</td><td>G</td><td>1556.0</td><td>EFF=UTR_5_PRIME(MODIFIER||622|||SPBC1E8.03c.1:exon:1|||transcript:SPBC1E8.03c.1|1|1)</td></tr>
<tr><td>I</td><td>3630676</td><td>.</td><td>T</td><td>A</td><td>1536.0</td><td>EFF=NON_SYNONYMOUS_CODING(MODERATE|MISSENSE|agA/agT|R222S||SPAC1142.03c.1:exon:1|||transcript:SPAC1142.03c.1|1|1)</td></tr>
<tr><td>II</td><td>1159694</td><td>.</td><td>A</td><td>T</td><td>1520.0</td><td>EFF=NON_SYNONYMOUS_CODING(MODERATE|MISSENSE|gaT/gaA|D385E||SPNCRNA.1417.1:exon:1|||transcript:SPNCRNA.1417.1|1|1|WARNING_TRANSCRIPT_INCOMPLETE),SYNONYMOUS_CODING(LOW|SILENT|ccA/ccT|P367||SPBC409.11.1:exon:1|||transcript:SPBC409.11.1|1|1)</td></tr>
<tr><td>II</td><td>276539</td><td>.</td><td>A</td><td>T</td><td>1496.0</td><td>EFF=NON_SYNONYMOUS_CODING(MODERATE|MISSENSE|gaA/gaT|E211D||SPBC800.11.1:exon:1|||transcript:SPBC800.11.1|1|1)</td></tr>
<tr><td>I</td><td>523883</td><td>.</td><td>G</td><td>A</td><td>1495.0</td><td>EFF=NON_SYNONYMOUS_CODING(MODERATE|MISSENSE|aGc/aAc|S213N||SPAC227.15.1:exon:1|||transcript:SPAC227.15.1|1|1)</td></tr>
</table>

SNVs

<table>
<tr><th>#CHROM</th><th>POS</th><th>ID</th><th>REF</th><th>ALT</th><th>QUAL</th><th></th></tr>
<tr><td>II</td><td>953201</td><td>.</td><td>A</td><td>G</td><td>1495.0</td><td>EFF=NON_SYNONYMOUS_CODING(MODERATE|MISSENSE|tTt/tCt|F328S||SPBC646.14c.1:exon:1|||transcript:SPBC646.14c.1|3|1)</td></tr>
<tr><td>III</td><td>963343</td><td>.</td><td>A</td><td>G</td><td>1487.0</td><td>EFF=SYNONYMOUS_CODING(LOW|SILENT|taT/taC|Y210||SPCC1742.01.1:exon:1|||transcript:SPCC1742.01.1|1|1)</td></tr>
<tr><td>II</td><td>699961</td><td>.</td><td>A</td><td>T</td><td>1475.0</td><td>EFF=INTERGENIC(MODIFIER||||||||||1)</td></tr>
<tr><td>II</td><td>3006137</td><td>.</td><td>A</td><td>T</td><td>1413.0</td><td>EFF=NON_SYNONYMOUS_CODING(MODERATE|MISSENSE|Tct/Act|S482T||SPBC15D4.01c.1:exon:1|||transcript:SPBC15D4.01c.1|3|1),UTR_3_PRIME(MODIFIER||747|||SPBC2D10.20.1:exon:1|||transcript:SPBC2D10.20.1|6|1)</td></tr>
<tr><td>II</td><td>2676336</td><td>.</td><td>C</td><td>T</td><td>1400.0</td><td>EFF=NON_SYNONYMOUS_CODING(MODERATE|MISSENSE|Gat/Aat|D8N||SPBC3E7.08c.1:exon:1|||transcript:SPBC3E7.08c.1|1|1)</td></tr>
<tr><td>II</td><td>2901509</td><td>.</td><td>G</td><td>T</td><td>1386.0</td><td>EFF=NON_SYNONYMOUS_CODING(MODERATE|MISSENSE|Cat/Aat|H367N||SPNCRNA.1562.1:exon:1|||transcript:SPNCRNA.1562.1|1|1|WARNING_TRANSCRIPT_INCOMPLETE),NON_SYNONYMOUS_CODING(MODERATE|MISSENSE|atG/atT|M89I||SPNCRNA.411.1:exon:1|||transcript:SPNCRNA.411.1|1|1|
WARNING_TRANSCRIPT_MULTIPLE_STOP_CODONS),NON_SYNONYMOUS_CODING(MODERATE|MISSENSE|tGt/tTt|C134F||SPNCRNA.1561.1:exon:1|||transcript:SPNCRNA.1561.1|1|1|WARNING_TRANSCRIPT_MULTIPLE_STOP_CODONS)</td></tr>
<tr><td>II</td><td>266718</td><td>.</td><td>A</td><td>C</td><td>1384.0</td><td>EFF=SYNONYMOUS_CODING(LOW|SILENT|cgA/cgC|R403||SPBC800.08.1:exon:1|||transcript:SPBC800.08.1|2|1),UTR_5_PRIME(MODIFIER||1904|||SPBC800.07c.1:exon:1|||transcript:SPBC800.07c.1|1|1)</td></tr>
<tr><td>III</td><td>1246664</td><td>.</td><td>G</td><td>T</td><td>1382.0</td><td>EFF=NON_SYNONYMOUS_CODING(MODERATE|MISSENSE|Gtc/Ttc|V468F||SPCC645.07.1:exon:1|||transcript:SPCC645.07.1|1|1)</td></tr>
<tr><td>II</td><td>1962458</td><td>.</td><td>T</td><td>A</td><td>1358.0</td><td>EFF=UTR_5_PRIME(MODIFIER||641|||SPBC1E8.03c.1:exon:1|||transcript:SPBC1E8.03c.1|1|1)</td></tr>
<tr><td>II</td><td>2689713</td><td>.</td><td>C</td><td>T</td><td>1355.0</td><td>EFF=NON_SYNONYMOUS_CODING(MODERATE|MISSENSE|Gtc/Atc|V212I||SPBC3E7.16c.1:exon:1|||transcript:SPBC3E7.16c.1|1|1)</td></tr>
<tr><td>I</td><td>1263527</td><td>.</td><td>A</td><td>G</td><td>1329.0</td><td>EFF=NON_SYNONYMOUS_CODING(MODERATE|MISSENSE|Aca/Gca|T26A||SPNCRNA.730.1:exon:1|||transcript:SPNCRNA.730.1|1|1|WARNING_TRANSCRIPT_MULTIPLE_STOP_CODONS)</td></tr>
<tr><td>II</td><td>700028</td><td>.</td><td>A</td><td>T</td><td>1317.0</td><td>EFF=SYNONYMOUS_CODING(LOW|SILENT|Tc/Ac|?594||SPNCRNA.1381.1:exon:1|||transcript:SPNCRNA.1381.1|1|1|WARNING_TRANSCRIPT_INCOMPLETE)</td></tr>
<tr><td>II</td><td>266780</td><td>.</td><td>T</td><td>A</td><td>1270.0</td><td>EFF=NON_SYNONYMOUS_CODING(MODERATE|MISSENSE|tTc/tAc|F424Y||SPBC800.08.1:exon:1|||transcript:SPBC800.08.1|2|1),UTR_5_PRIME(MODIFIER||1966|||SPBC800.07c.1:exon:1|||transcript:SPBC800.07c.1|1|1)</td></tr>
<tr><td>I</td><td>3204845</td><td>.</td><td>G</td><td>A</td><td>1265.0</td><td>EFF=NON_SYNONYMOUS_CODING(MODERATE|MISSENSE|cGa/cAa|R162Q||SPAC1486.06.1:five_prime_UTR:1|||transcript:SPAC1486.06.1|2|1)</td></tr>
<tr><td>II</td><td>700024</td><td>.</td><td>T</td><td>A</td><td>1241.0</td><td>EFF=INTERGENIC(MODIFIER||||||||||1)</td></tr>
<tr><td>II</td><td>967094</td><td>.</td><td>A</td><td>G</td><td>1225.0</td><td>EFF=SYNONYMOUS_CODING(LOW|SILENT|gaA/gaG|E128||SPBP35G2.02.1:exon:1|||transcript:SPBP35G2.02.1|1|1)</td></tr>
<tr><td>I</td><td>2104097</td><td>.</td><td>C</td><td>A</td><td>1214.0</td><td>EFF=UTR_3_PRIME(MODIFIER||100|||SPAC631.01c.1:exon:1|||transcript:SPAC631.01c.1|3|1),UTR_3_PRIME(MODIFIER||143|||SPAC25A8.02.1:exon:1|||transcript:SPAC25A8.02.1|1|1)</td></tr>
<tr><td>II</td><td>699952</td><td>.</td><td>G</td><td>T</td><td>1202.0</td><td>EFF=INTERGENIC(MODIFIER||||||||||1)</td></tr>
<tr><td>III</td><td>227730</td><td>.</td><td>A</td><td>T</td><td>1184.0</td><td>EFF=NON_SYNONYMOUS_CODING(MODERATE|MISSENSE|gTt/gAt|V224D||SPCC548.06c.1:exon:1|||transcript:SPCC548.06c.1|1|1)</td></tr>
<tr><td>II</td><td>869806</td><td>.</td><td>G</td><td>C</td><td>1181.0</td><td>EFF=NON_SYNONYMOUS_CODING(MODERATE|MISSENSE|Cat/Gat|H42D||SPBC713.02c.1:exon:1|||transcript:SPBC713.02c.1|3|1)</td></tr>
<tr><td>II</td><td>699951</td><td>.</td><td>T</td><td>G</td><td>1179.0</td><td>EFF=INTERGENIC(MODIFIER||||||||||1)</td></tr>
<tr><td>I</td><td>2104091</td><td>.</td><td>G</td><td>T</td><td>1171.0</td><td>EFF=UTR_3_PRIME(MODIFIER||106|||SPAC631.01c.1:exon:1|||transcript:SPAC631.01c.1|3|1),UTR_3_PRIME(MODIFIER||137|||SPAC25A8.02.1:exon:1|||transcript:SPAC25A8.02.1|1|1)</td></tr>
<tr><td>II</td><td>2444543</td><td>.</td><td>T</td><td>C</td><td>1160.0</td><td>EFF=NON_SYNONYMOUS_CODING(MODERATE|MISSENSE|gTt/gCt|V72A||SPBC12C2.13c.1:exon:1|||transcript:SPBC12C2.13c.1|1|1),SYNONYMOUS_STOP(LOW|SILENT|taA/taG|*96||SPNCRNA.1514.1:exon:1|||transcript:SPNCRNA.1514.1|1|1|WARNING_TRANSCRIPT_INCOMPLETE)</td></tr>
<tr><td>II</td><td>700020</td><td>.</td><td>C</td><td>T</td><td>1124.0</td><td>EFF=INTERGENIC(MODIFIER||||||||||1)</td></tr>
<tr><td>II</td><td>700019</td><td>.</td><td>T</td><td>A</td><td>1123.0</td><td>EFF=INTERGENIC(MODIFIER||||||||||1)</td></tr>
<tr><td>II</td><td>2806053</td><td>.</td><td>G</td><td>A</td><td>1120.0</td><td>EFF=NON_SYNONYMOUS_CODING(MODERATE|MISSENSE|Gga/Aga|G24R||SPNCRNA.1550.1:exon:1|||transcript:SPNCRNA.1550.1|1|1|WARNING_TRANSCRIPT_INCOMPLETE)</td></tr>
<tr><td>II</td><td>700021</td><td>.</td><td>A</td><td>C</td><td>1117.0</td><td>EFF=INTERGENIC(MODIFIER||||||||||1)</td></tr>
<tr><td>II</td><td>2007715</td><td>.</td><td>A</td><td>G</td><td>1099.0</td><td>EFF=INTERGENIC(MODIFIER||||||||||1)</td></tr>
<tr><td>II</td><td>962880</td><td>.</td><td>C</td><td>G</td><td>1095.0</td><td>EFF=UTR_5_PRIME(MODIFIER||365|||SPBP35G2.16c.1:exon:1|||transcript:SPBP35G2.16c.1|1|1)</td></tr>
<tr><td>III</td><td>1772337</td><td>.</td><td>G</td><td>A</td><td>1046.0</td><td>EFF=SYNONYMOUS_CODING(LOW|SILENT|agG/agA|R241||SPCC1442.03.1:exon:1|||transcript:SPCC1442.03.1|2|1)</td></tr>
<tr><td>III</td><td>1260881</td><td>.</td><td>G</td><td>A</td><td>1011.0</td><td>EFF=UTR_3_PRIME(MODIFIER||82|||SPCC645.13.1:five_prime_UTR:1|||transcript:SPCC645.13.1|2|1)</td></tr>
<tr><td>III</td><td>1260882</td><td>.</td><td>G</td><td>A</td><td>999.0</td><td>EFF=UTR_3_PRIME(MODIFIER||83|||SPCC645.13.1:five_prime_UTR:1|||transcript:SPCC645.13.1|2|1)</td></tr>
<tr><td>II</td><td>2044929</td><td>.</td><td>A</td><td>T</td><td>995.0</td><td>EFF=NON_SYNONYMOUS_CODING(MODERATE|MISSENSE|Aac/Tac|N290Y||SPNCRNA.1483.1:exon:1|||transcript:SPNCRNA.1483.1|1|1|WARNING_TRANSCRIPT_MULTIPLE_STOP_CODONS)</td></tr>
<tr><td>II</td><td>2436580</td><td>.</td><td>C</td><td>G</td><td>987.0</td><td>EFF=SYNONYMOUS_CODING(LOW|SILENT|tcC/tcG|S315||SPBC21D10.06c.1:exon:1|||transcript:SPBC21D10.06c.1|1|1)</td></tr>
<tr><td>I</td><td>2506044</td><td>.</td><td>T</td><td>A</td><td>933.0</td><td>EFF=INTERGENIC(MODIFIER||||||||||1)</td></tr>
<tr><td>II</td><td>1697124</td><td>.</td><td>C</td><td>T</td><td>904.0</td><td>EFF=UTR_3_PRIME(MODIFIER||283|||SPBC19C2.10.1:exon:1|||transcript:SPBC19C2.10.1|2|1)</td></tr>
<tr><td>I</td><td>943745</td><td>.</td><td>T</td><td>C</td><td>890.0</td><td>EFF=NON_SYNONYMOUS_CODING(MODERATE|MISSENSE|atA/atG|I709M||SPAC1687.22c.1:exon:1|||transcript:SPAC1687.22c.1|2|1)</td></tr>
<tr><td>II</td><td>1716326</td><td>.</td><td>G</td><td>T</td><td>824.0</td><td>EFF=INTERGENIC(MODIFIER||||||||||1)</td></tr>
<tr><td>II</td><td>4400666</td><td>.</td><td>A</td><td>C</td><td>824.0</td><td>EFF=NON_SYNONYMOUS_CODING(MODERATE|MISSENSE|Tgg/Ggg|W728G||SPBC1289.10c.1:exon:1|||transcript:SPBC1289.10c.1|1|1)</td></tr>
<tr><td>II</td><td>1683324</td><td>.</td><td>T</td><td>G</td><td>817.0</td><td>EFF=NON_SYNONYMOUS_CODING(MODERATE|MISSENSE|gTa/gGa|V155G||SPBC19C2.05.1:exon:1|||transcript:SPBC19C2.05.1|1|1)</td></tr>
<tr><td>II</td><td>2437627</td><td>.</td><td>C</td><td>T</td><td>795.0</td><td>EFF=SYNONYMOUS_CODING(LOW|SILENT|gtC/gtT|V664||SPBC21D10.06c.1:exon:1|||transcript:SPBC21D10.06c.1|1|1)</td></tr>
<tr><td>III</td><td>284657</td><td>.</td><td>C</td><td>A</td><td>787.0</td><td>EFF=UTR_5_PRIME(MODIFIER||186|||SPCC553.10.1:exon:1|||transcript:SPCC553.10.1|1|1)</td></tr>
<tr><td>III</td><td>1168328</td><td>.</td><td>T</td><td>C</td><td>771.0</td><td>EFF=INTERGENIC(MODIFIER||||||||||1)</td></tr>
<tr><td>II</td><td>4538155</td><td>.</td><td>T</td><td>G</td><td>736.0</td><td>EFF=INTERGENIC(MODIFIER||||||||||1)</td></tr>
<tr><td>II</td><td>1678693</td><td>.</td><td>T</td><td>A</td><td>671.0</td><td>EFF=INTERGENIC(MODIFIER||||||||||1)</td></tr>
<tr><td>II</td><td>1716318</td><td>.</td><td>A</td><td>G</td><td>670.0</td><td>EFF=INTERGENIC(MODIFIER||||||||||1)</td></tr>
<tr><td>I</td><td>4442714</td><td>.</td><td>G</td><td>A</td><td>666.0</td><td>EFF=NON_SYNONYMOUS_CODING(MODERATE|MISSENSE|aCt/aTt|T69I||SPNCRNA.988.1:exon:1|||transcript:SPNCRNA.988.1|1|1|WARNING_TRANSCRIPT_INCOMPLETE)</td></tr>
<tr><td>II</td><td>1716320</td><td>.</td><td>T</td><td>G</td><td>665.0</td><td>EFF=INTERGENIC(MODIFIER||||||||||1)</td></tr>
<tr><td>I</td><td>2605764</td><td>.</td><td>T</td><td>A</td><td>650.0</td><td>EFF=UTR_5_PRIME(MODIFIER||421|||SPAC823.14.1:exon:1|||transcript:SPAC823.14.1|1|1)</td></tr>
<tr><td>II</td><td>689794</td><td>.</td><td>T</td><td>C</td><td>630.0</td><td>EFF=SYNONYMOUS_CODING(LOW|SILENT|tcT/tcC|S693||SPBPJ4664.02.1:exon:1|||transcript:SPBPJ4664.02.1|1|1)</td></tr>
<tr><td>I</td><td>2747078</td><td>.</td><td>A</td><td>G</td><td>624.0</td><td>EFF=INTRON(MODIFIER|||||SPAC6F6.08c.1:exon:1|||transcript:SPAC6F6.08c.1|3|1)</td></tr>
<tr><td>II</td><td>4532088</td><td>.</td><td>G</td><td>T</td><td>595.0</td><td>EFF=NON_SYNONYMOUS_CODING(MODERATE|MISSENSE|gGt/gTt|G1735V||SPBCPT2R1.08c.1:exon:1|||transcript:SPBCPT2R1.08c.1|1|1)</td></tr>
<tr><td>II</td><td>1155903</td><td>.</td><td>T</td><td>A</td><td>589.0</td><td>EFF=INTERGENIC(MODIFIER||||||||||1)</td></tr>
<tr><td>II</td><td>689487</td><td>.</td><td>A</td><td>C</td><td>547.0</td><td>EFF=NON_SYNONYMOUS_CODING(MODERATE|MISSENSE|tAt/tCt|Y591S||SPBPJ4664.02.1:exon:1|||transcript:SPBPJ4664.02.1|1|1)</td></tr>
<tr><td>II</td><td>1553976</td><td>.</td><td>A</td><td>T</td><td>544.0</td><td>EFF=STOP_LOST(HIGH|MISSENSE|taA/taT|*156Y||SPNCRNA.1442.1:exon:1|||transcript:SPNCRNA.1442.1|1|1|WARNING_TRANSCRIPT_INCOMPLETE)</td></tr>
</table>

<table>
<tr><th colspan="7">SNVs</th></tr>
<tr><th>#CHROM</th><th>POS</th><th>ID</th><th>REF</th><th>ALT</th><th>QUAL</th><th></th></tr>
<tr><td>I</td><td>3548913</td><td>.</td><td>T</td><td>A</td><td>507.0</td><td>EFF=INTERGENIC(MODIFIER||||||||||1)</td></tr>
<tr><td>II</td><td>689485</td><td>.</td><td>A</td><td>T</td><td>480.0</td><td>EFF=NON_SYNONYMOUS_CODING(MODERATE|MISSENSE|agA/agT|R590S||SPBPJ4664.02.1:exon:1|||transcript:SPBPJ4664.02.1|1|1)</td></tr>
<tr><td>II</td><td>4538038</td><td>.</td><td>A</td><td>G</td><td>471.0</td><td>EFF=INTERGENIC(MODIFIER||||||||||1)</td></tr>
<tr><td>II</td><td>4537844</td><td>.</td><td>T</td><td>C</td><td>463.0</td><td>SF=0,1</td></tr>
<tr><td>II</td><td>4538052</td><td>.</td><td>A</td><td>G</td><td>461.0</td><td>EFF=INTERGENIC(MODIFIER||||||||||1)</td></tr>
<tr><td>I</td><td>3538450</td><td>.</td><td>A</td><td>T</td><td>440.0</td><td>EFF=UTR_3_PRIME(MODIFIER||191|||SPAC1B1.01.1:exon:1|||transcript:SPAC1B1.01.1|2|1)</td></tr>
<tr><td>III</td><td>720001</td><td>.</td><td>C</td><td>T</td><td>361.0</td><td>EFF=INTERGENIC(MODIFIER||||||||||1)</td></tr>
<tr><td>I</td><td>3115841</td><td>.</td><td>C</td><td>A</td><td>345.0</td><td>EFF=UTR_3_PRIME(MODIFIER||226|||SPAC688.04c.1:exon:1|||transcript:SPAC688.04c.1|1|1)</td></tr>
<tr><td>I</td><td>1664907</td><td>.</td><td>T</td><td>A</td><td>317.0</td><td>EFF=INTERGENIC(MODIFIER||||||||||1)</td></tr>
<tr><td>I</td><td>3992214</td><td>.</td><td>C</td><td>A</td><td>288.0</td><td>EFF=NON_SYNONYMOUS_CODING(MODERATE|MISSENSE|aaC/aaA|N291K||SPNCRNA.954.1:exon:1|||transcript:SPNCRNA.954.1|1|1|WARNING_TRANSCRIPT_INCOMPLETE),NON_SYNONYMOUS_CODING(MODERATE|MISSENSE|tgG/tgT|W77C||SPAPB15E9.02c.1:exon:1|||transcript:SPAPB15E9.02c.1|1|1)</td></tr>
<tr><td>I</td><td>1465627</td><td>.</td><td>G</td><td>A</td><td>251.0</td><td>EFF=INTERGENIC(MODIFIER||||||||||1)</td></tr>
<tr><td>I</td><td>2245490</td><td>.</td><td>G</td><td>C</td><td>205.0</td><td>EFF=INTERGENIC(MODIFIER||||||||||1)</td></tr>
</table>

<table>
<tr><th colspan="6">Indels</th></tr>
<tr><th>#CHROM</th><th>POS</th><th>REF</th><th>ALT</th><th>QUAL</th><th></th></tr>
<tr><td>I</td><td>10859</td><td>GTT</td><td>G</td><td>0.0</td><td>EFF=INTERGENIC(MODIFIER||||||||||1)</td></tr>
<tr><td>I</td><td>19694</td><td>T</td><td>TA</td><td>184.97</td><td>EFF=INTERGENIC(MODIFIER||||||||||1)</td></tr>
<tr><td>I</td><td>101871</td><td>A</td><td>AG</td><td>1276.97</td><td>EFF=FRAME_SHIFT(HIGH||gcc/gcCc|A558A?||SPAC1F8.07c.1:exon:1|||transcript:SPAC1F8.07c.1|1|1),SPLICE_SITE_REGION(LOW|||||SPAC1F8.07c.1:exon:1|||transcript:SPAC1F8.07c.1|1|1)</td></tr>
<tr><td>I</td><td></td><td>C</td><td>CT</td><td>2397.97</td><td>EFF=NON_SYNONYMOUS_CODING(MODERATE||cgtgatcNN/c79.793NN|RD?1886???||SPAC212.11.1:exon:1|||transcript:SPAC212.11.1||1|WARNING_TRANSCRIPT_INCOMPLETE)</td></tr>
<tr><td>I</td><td>470378</td><td>AC</td><td>A</td><td>1989.97</td><td>EFF=UTR_3_PRIME(MODIFIER||204|||SPAC24H6.09.1:exon:1|||transcript:SPAC24H6.09.1|2|1)</td></tr>
<tr><td>I</td><td>470390</td><td>AC</td><td>A</td><td>1870.97</td><td>EFF=UTR_3_PRIME(MODIFIER||192|||SPAC24H6.09.1:exon:1|||transcript:SPAC24H6.09.1|2|1)</td></tr>
<tr><td>I</td><td>482737</td><td>A</td><td>AG</td><td>2752.97</td><td>EFF=UTR_3_PRIME(MODIFIER||709|||SPAC24H6.04.1:exon:1|||transcript:SPAC24H6.04.1|1|1)</td></tr>
<tr><td>I</td><td>526170</td><td>G</td><td>GA</td><td>1908.97</td><td>EFF=UTR_3_PRIME(MODIFIER||304|||SPAC227.15.1:exon:1|||transcript:SPAC227.15.1|1|1)</td></tr>
<tr><td>I</td><td>554527</td><td>A</td><td>AT</td><td>326.97</td><td>EFF=INTERGENIC(MODIFIER||||||||||1)</td></tr>
<tr><td>I</td><td>616208</td><td>G</td><td>GT</td><td>2061.97</td><td>EFF=FRAME_SHIFT(HIGH||aca/aAca|T214N?||SPAC1F3.02c.1:exon:1|||transcript:SPAC1F3.02c.1|1|1)</td></tr>
<tr><td>I</td><td>660056</td><td>TG</td><td>T</td><td>764.97</td><td>EFF=UTR_5_PRIME(MODIFIER||1225|||SPAC1D4.11c.1:exon:1|||transcript:SPAC1D4.11c.1|1|1)</td></tr>
<tr><td>I</td><td>670032</td><td>C</td><td>CA</td><td>2032.97</td><td>EFF=INTERGENIC(MODIFIER||||||||||1)</td></tr>
<tr><td>I</td><td>682993</td><td>TC</td><td>T</td><td>1689.97</td><td>EFF=FRAME_SHIFT(HIGH||-/-|-41||SPAC22F3.11c.1:exon:1|||transcript:SPAC22F3.11c.1|1|1)</td></tr>
<tr><td>I</td><td>862962</td><td>C</td><td>CT</td><td>3637.97</td><td>EFF=INTRON(MODIFIER|||||SPAC23G3.02c.1:exon:1|||transcript:SPAC23G3.02c.1|1|1)</td></tr>
<tr><td>I</td><td>959770</td><td>AT</td><td>A</td><td>1278.97</td><td>EFF=INTERGENIC(MODIFIER||||||||||1)</td></tr>
<tr><td>I</td><td>960025</td><td>G</td><td>GT</td><td>96.97</td><td>EFF=UTR_5_PRIME(MODIFIER||35|||SPAC222.09.1:exon:1|||transcript:SPAC222.09.1|1|1)</td></tr>
<tr><td>I</td><td>995016</td><td>GT</td><td>G</td><td>1016.97</td><td>EFF=FRAME_SHIFT(HIGH||-/-|-377||SPNCRNA.703.1:exon:1|||transcript:SPNCRNA.703.1|1|1|WARNING_TRANSCRIPT_INCOMPLETE),UTR_3_PRIME(MODIFIER||459|||SPAC821.08c.1:exon:1|||transcript:SPAC821.08c.1|1|1)</td></tr>
<tr><td>I</td><td>1048635</td><td>G</td><td>GA</td><td>223.97</td><td>EFF=FRAME_SHIFT(HIGH||ttc/ttTc|F100F?||SPNCRNA.708.1:exon:1|||transcript:SPNCRNA.708.1|1|1|WARNING_TRANSCRIPT_MULTIPLE_STOP_CODONS)</td></tr>
<tr><td>I</td><td>1274803</td><td>T</td><td>TA</td><td>1552.97</td><td>EFF=INTERGENIC(MODIFIER||||||||||1)</td></tr>
<tr><td>I</td><td>1424708</td><td>CA</td><td>C</td><td>2861.97</td><td>EFF=FRAME_SHIFT(HIGH||-/-|-232||SPAC3A12.04c.1:exon:1|||transcript:SPAC3A12.04c.1|4|1)</td></tr>
<tr><td>I</td><td>1465713</td><td>CT</td><td>C</td><td>1183.97</td><td>EFF=INTERGENIC(MODIFIER||||||||||1)</td></tr>
<tr><td>I</td><td>1509277</td><td>C</td><td>CT</td><td>0.0</td><td>EFF=INTERGENIC(MODIFIER||||||||||1)</td></tr>
<tr><td>I</td><td>1625092</td><td>T</td><td>TC</td><td>1942.97</td><td>EFF=FRAME_SHIFT(HIGH||gag/gGag|E1560G?||SPAP27G11.10c.1:exon:1|||transcript:SPAP27G11.10c.1|2|1)</td></tr>
<tr><td>I</td><td>1936294</td><td>G</td><td>GA</td><td>1534.97</td><td>EFF=INTRON(MODIFIER|||||SPAC3H1.04c.1:exon:1|||transcript:SPAC3H1.04c.1|1|1)</td></tr>
<tr><td>I</td><td>1952313</td><td>A</td><td>AT</td><td>1606.97</td><td>EFF=FRAME_SHIFT(HIGH||ata/aAta|I428N?||SPNCRNA.777.1:exon:1|||transcript:SPNCRNA.777.1|1|1|WARNING_TRANSCRIPT_INCOMPLETE)</td></tr>
<tr><td>I</td><td>2139856</td><td>G</td><td>GT</td><td>86.97</td><td>EFF=INTERGENIC(MODIFIER||||||||||1)</td></tr>
<tr><td>I</td><td>2154491</td><td>T</td><td>TA</td><td>2841.97</td><td>EFF=FRAME_SHIFT(HIGH||cta/ctTa|L7L?||SPNCRNA.800.1:exon:1|||transcript:SPNCRNA.800.1|1|1|WARNING_TRANSCRIPT_INCOMPLETE)</td></tr>
<tr><td>I</td><td>2288240</td><td>CG</td><td>C</td><td>2592.97</td><td>EFF=UTR_3_PRIME(MODIFIER||52|||SPAC4G9.19.1:exon:1|||transcript:SPAC4G9.19.1|1|1)</td></tr>
<tr><td>I</td><td>2290217</td><td>C</td><td>CTAGTA</td><td>2266.97</td><td>EFF=INTERGENIC(MODIFIER||||||||||1)</td></tr>
<tr><td>I</td><td>2343703</td><td>G</td><td>GA</td><td>2152.97</td><td>EFF=FRAME_SHIFT(HIGH||act/actT|T2T?||SPAC17G8.01c.1:exon:1|||transcript:SPAC17G8.01c.1|2|1),SPLICE_SITE_REGION(LOW|||||SPAC17G8.01c.1:exon:1|||transcript:SPAC17G8.01c.1|2|1)</td></tr>
<tr><td>I</td><td>2421576</td><td>AC</td><td>A</td><td>2625.97</td><td>EFF=UTR_3_PRIME(MODIFIER||214|||SPAC6B12.07c.1:exon:1|||transcript:SPAC6B12.07c.1|1|1)</td></tr>
</table>

Indels

<table>
<tr><th>#CHROM</th><th>POS</th><th>REF</th><th>ALT</th><th>QUAL</th><th></th></tr>
<tr><td>I</td><td>2499149</td><td>AT</td><td>A</td><td>1046.97</td><td>EFF=FRAME_SHIFT(HIGH||-/-|-39||SPAC23H3.04.1:exon:1|||transcript:SPAC23H3.04.1|1|1)</td></tr>
<tr><td>I</td><td>2507462</td><td>T</td><td>TA</td><td>2421.97</td><td>EFF=UTR_5_PRIME(MODIFIER||157|||SPAC23H3.08c.1:exon:1|||transcript:SPAC23H3.08c.1|1|1)</td></tr>
<tr><td>I</td><td>2510060</td><td>T</td><td>TA</td><td>1587.97</td><td>EFF=INTERGENIC(MODIFIER||||||||||1)</td></tr>
<tr><td>I</td><td>2588021</td><td>C</td><td>CA</td><td>2266.97</td><td>EFF=FRAME_SHIFT(HIGH||aca/acAa|T80T?||SPAC823.04.1:exon:1|||transcript:SPAC823.04.1|2|1)</td></tr>
<tr><td>I</td><td>2588066</td><td>C</td><td>CA</td><td>1487.97</td><td>EFF=FRAME_SHIFT(HIGH||aaa/Aaaa|K95K?||SPAC823.04.1:exon:1|||transcript:SPAC823.04.1|3|1)</td></tr>
<tr><td>I</td><td>2594263</td><td>C</td><td>CT</td><td>2198.97</td><td>EFF=INTERGENIC(MODIFIER||||||||||1)</td></tr>
<tr><td>I</td><td>2594324</td><td>T</td><td>TA</td><td>2841.97</td><td>EFF=INTERGENIC(MODIFIER||||||||||1)</td></tr>
<tr><td>I</td><td>2594351</td><td>T</td><td>TA</td><td>2442.97</td><td>EFF=INTERGENIC(MODIFIER||||||||||1)</td></tr>
<tr><td>I</td><td>2594391</td><td>A</td><td>AT</td><td>2474.97</td><td>EFF=INTERGENIC(MODIFIER||||||||||1)</td></tr>
<tr><td>I</td><td>2605860</td><td>GT</td><td>G</td><td>2631.97</td><td>EFF=UTR_5_PRIME(MODIFIER||324|||SPAC823.14.1:exon:1|||transcript:SPAC823.14.1|1|1)</td></tr>
<tr><td>I</td><td>2607669</td><td>A</td><td>AT</td><td>2206.97</td><td>EFF=FRAME_SHIFT(HIGH||aat/aaAt|N116K?||SPNCRNA.841.1:exon:1|||transcript:SPNCRNA.841.1|1|1|WARNING_TRANSCRIPT_INCOMPLETE),INTRAGENIC(MODIFIER||||ppa1||||1),UTR_5_PRIME(MODIFIER||433|||SPAC823.15.1:five_prime_UTR:1|||transcript:SPAC823.15.1|1|1)</td></tr>
<tr><td>I</td><td>2683902</td><td>TC</td><td>T</td><td>1992.97</td><td>EFF=FRAME_SHIFT(HIGH||-/-|-456||SPNCRNA.844.1:exon:1|||transcript:SPNCRNA.844.1|1|1|WARNING_TRANSCRIPT_MULTIPLE_STOP_CODONS)</td></tr>
<tr><td>I</td><td>2751236</td><td>TA</td><td>T</td><td>121.97</td><td>EFF=INTERGENIC(MODIFIER||||||||||1)</td></tr>
<tr><td>I</td><td>2759871</td><td>T</td><td>TC</td><td>1827.97</td><td>EFF=FRAME_SHIFT(HIGH||tct/tCct|S361S?||SPNCRNA.851.1:exon:1|||transcript:SPNCRNA.851.1|1|1|WARNING_TRANSCRIPT_MULTIPLE_STOP_CODONS)</td></tr>
<tr><td>I</td><td>2949974</td><td>GAT</td><td>G</td><td>438.97</td><td>EFF=UTR_3_PRIME(MODIFIER||247|||SPAPB24D3.01.1:exon:1|||transcript:SPAPB24D3.01.1|1|1)</td></tr>
<tr><td>I</td><td>2958937</td><td>GAA</td><td>G</td><td>417.97</td><td>EFF=FRAME_SHIFT(HIGH||-/-|-712||SPNCRNA.865.1:exon:1|||transcript:SPNCRNA.865.1|1|1|WARNING_TRANSCRIPT_INCOMPLETE),UTR_5_PRIME(MODIFIER||12|||SPAPB24D3.07c.1:exon:1|||transcript:SPAPB24D3.07c.1|1|1)</td></tr>
<tr><td>I</td><td>3125118</td><td>A</td><td>AT</td><td>2804.97</td><td>EFF=FRAME_SHIFT(HIGH||ata/aTta|I639I?||SPAC688.08.1:exon:1|||transcript:SPAC688.08.1|2|1)</td></tr>
<tr><td>I</td><td>3178494</td><td>C</td><td>CT</td><td>2238.97</td><td>EFF=FRAME_SHIFT(HIGH||ttc/Tttc|F189F?||SPNCRNA.893.1:exon:1|||transcript:SPNCRNA.893.1|1|1|WARNING_TRANSCRIPT_INCOMPLETE)</td></tr>
<tr><td>I</td><td>3197528</td><td>A</td><td>AG</td><td>1041.97</td><td>EFF=FRAME_SHIFT(HIGH||agg/aGgg|R167R?||SPAC1486.05.1:exon:1|||transcript:SPAC1486.05.1|2|1),SPLICE_SITE_REGION(LOW||||SPAC1486.05.1:exon:1|||transcript:SPAC1486.05.1|2|1)</td></tr>
<tr><td>I</td><td>3318900</td><td>T</td><td>TA</td><td>1042.97</td><td>EFF=UTR_5_PRIME(MODIFIER||269|||SPAPB1E7.11c.1:five_prime_UTR:1|||transcript:SPAPB1E7.11c.1|1|1)</td></tr>
<tr><td>I</td><td>3450130</td><td>GT</td><td>G</td><td>1672.97</td><td>EFF=FRAME_SHIFT(HIGH||-/-|-755||SPAC3A11.09.1:exon:1|||transcript:SPAC3A11.09.1|2|1)</td></tr>
<tr><td>I</td><td>3460318</td><td>T</td><td>TC</td><td>1709.97</td><td>EFF=FRAME_SHIFT(HIGH||gga/ggGa|G365G?||SPAC3A11.06.1:exon:1|||transcript:SPAC3A11.06.1|6|1)</td></tr>
<tr><td>I</td><td>3538413</td><td>A</td><td>AT</td><td>1452.97</td><td>EFF=UTR_3_PRIME(MODIFIER||155|||SPAC1B1.01.1:exon:1|||transcript:SPAC1B1.01.1|2|1)</td></tr>
<tr><td>I</td><td>3538428</td><td>G</td><td>GT</td><td>1340.97</td><td>EFF=UTR_3_PRIME(MODIFIER||170|||SPAC1B1.01.1:exon:1|||transcript:SPAC1B1.01.1|2|1)</td></tr>
<tr><td>I</td><td>3538436</td><td>T</td><td>TA</td><td>1193.97</td><td>EFF=UTR_3_PRIME(MODIFIER||178|||SPAC1B1.01.1:exon:1|||transcript:SPAC1B1.01.1|2|1)</td></tr>
<tr><td>I</td><td>3538449</td><td>T</td><td>TTA</td><td>1076.97</td><td>EFF=UTR_3_PRIME(MODIFIER||191|||SPAC1B1.01.1:exon:1|||transcript:SPAC1B1.01.1|2|1)</td></tr>
<tr><td>I</td><td>3538456</td><td>A</td><td>AAT</td><td>1124.97</td><td>EFF=UTR_3_PRIME(MODIFIER||198|||SPAC1B1.01.1:exon:1|||transcript:SPAC1B1.01.1|2|1)</td></tr>
<tr><td>I</td><td>3548899</td><td>AAAAAAAAAAAGAAT</td><td>A</td><td>552.97</td><td>EFF=INTERGENIC(MODIFIER||||||||||1)</td></tr>
<tr><td>I</td><td>3576756</td><td>T</td><td>TTCTTCTC</td><td>741.97</td><td>EFF=INTERGENIC(MODIFIER||||||||||1)</td></tr>
<tr><td>I</td><td>3576929</td><td>C</td><td>CT</td><td>221.97</td><td>EFF=UTR_5_PRIME(MODIFIER||179|||SPAC17A2.11.1:exon:1|||transcript:SPAC17A2.11.1|1|1)</td></tr>
<tr><td>I</td><td>3578638</td><td>AT</td><td>A</td><td>0.0</td><td>EFF=FRAME_SHIFT(HIGH||-/-|-897||SPNCRNA.918.1:exon:1|||transcript:SPNCRNA.918.1|1|1|WARNING_TRANSCRIPT_INCOMPLETE),UTR_3_PRIME(MODIFIER||877|||SPAC17A2.11.1:exon:1|||transcript:SPAC17A2.11.1|1|1)</td></tr>
<tr><td>I</td><td>3665610</td><td>G</td><td>GC</td><td>2126.97</td><td>EFF=INTERGENIC(MODIFIER||||||||||1)</td></tr>
<tr><td>I</td><td>3730369</td><td>A</td><td>AT</td><td>1534.97</td><td>EFF=INTRON(MODIFIER||||SPAC15E1.08.1:exon:1|||transcript:SPAC15E1.08.1|2|1)</td></tr>
<tr><td>I</td><td>3855790</td><td>GT</td><td>G</td><td>2414.97</td><td>EFF=FRAME_SHIFT(HIGH||-/-|-668||SPAC1071.01c.1:exon:1|||transcript:SPAC1071.01c.1|1|1)</td></tr>
<tr><td>I</td><td>3940025</td><td>G</td><td>GT</td><td>544.97</td><td>EFF=FRAME_SHIFT(HIGH||aac/aaAc|N104K?||SPNCRNA.949.1:exon:1|||transcript:SPNCRNA.949.1|1|1|WARNING_TRANSCRIPT_INCOMPLETE),UTR_5_PRIME(MODIFIER||237|||SPAC2F3.09.1:exon:1|||transcript:SPAC2F3.09.1|1|1)</td></tr>
<tr><td>I</td><td>4055052</td><td>T</td><td>TA</td><td>99.97</td><td>EFF=INTRON(MODIFIER||||SPAC19G12.07c.1:exon:1|||transcript:SPAC19G12.07c.1|2|1)</td></tr>
<tr><td>I</td><td>4261340</td><td>C</td><td>CT</td><td>461.97</td><td>EFF=INTERGENIC(MODIFIER||||||||||1)</td></tr>
<tr><td>I</td><td>4276890</td><td>G</td><td>GT</td><td>0.0</td><td>EFF=UTR_3_PRIME(MODIFIER||105|||SPAC2C4.09.1:exon:1|||transcript:SPAC2C4.09.1|3|1)</td></tr>
<tr><td>I</td><td>4304169</td><td>G</td><td>GA</td><td>1140.97</td><td>EFF=INTERGENIC(MODIFIER||||||||||1)</td></tr>
<tr><td>I</td><td>4374342</td><td>AG</td><td>A</td><td>2563.97</td><td>EFF=INTRON(MODIFIER||||SPAC23D3.14c.1:exon:1|||transcript:SPAC23D3.14c.1|2|1)</td></tr>
<tr><td>I</td><td>4407494</td><td>T</td><td>TG</td><td>2501.97</td><td>EFF=FRAME_SHIFT(HIGH||cgc/cgcC|R590R?||SPAC29E6.03c.1:exon:1|||transcript:SPAC29E6.03c.1|2|1),UTR_3_PRIME(MODIFIER||1559|||SPAC29E6.02.1:exon:1|||transcript:SPAC29E6.02.1|5|1)</td></tr>
<tr><td>I</td><td>4410191</td><td>CG</td><td>C</td><td>2553.97</td><td>EFF=FRAME_SHIFT(HIGH||-/-|-140||SPAC29E6.04.1:exon:1|||transcript:SPAC29E6.04.1|1|1),SPLICE_SITE_REGION(LOW||||SPAC29E6.04.1:exon:1|||transcript:SPAC29E6.04.1|1|1),UTR_3_PRIME(MODIFIER||413|||SPAC29E6.05c.1:exon:1|||transcript:SPAC29E6.05c.1|2|1)</td></tr>
<tr><td>I</td><td>4431856</td><td>G</td><td>GA</td><td>2287.97</td><td>EFF=INTERGENIC(MODIFIER||||||||||1)</td></tr>
<tr><td>I</td><td>4431868</td><td>C</td><td>CA</td><td>2151.97</td><td>EFF=INTRAGENIC(MODIFIER||||sfp1||||1),UTR_3_PRIME(MODIFIER||435|||SPAC16.05c.1:exon:1|||transcript:SPAC16.05c.1|1|1)</td></tr>
<tr><td>I</td><td>4455149</td><td>G</td><td>GA</td><td>88.97</td><td>EFF=UTR_5_PRIME(MODIFIER||79|||SPAC9E9.09c.1:exon:1|||transcript:SPAC9E9.09c.1|1|1)</td></tr>
<tr><td>I</td><td>4721875</td><td>G</td><td>GA</td><td>929.97</td><td>EFF=FRAME_SHIFT(HIGH||gaa/gAaa|E208E?||SPNCRNA.1018.1:exon:1|||transcript:SPNCRNA.1018.1|1|1|WARNING_TRANSCRIPT_MULTIPLE_STOP_CODONS),UTR_3_PRIME(MODIFIER||472|||SPAC1834.13.1:exon:1|||transcript:SPAC1834.13.1|1|1)</td></tr>
<tr><td>I</td><td>4764253</td><td>G</td><td>GA</td><td>110.97</td><td>EFF=INTERGENIC(MODIFIER||||||||||1)</td></tr>
<tr><td>I</td><td>5055030</td><td>GA</td><td>G</td><td>1321.97</td><td>EFF=INTERGENIC(MODIFIER||||||||||1)</td></tr>
<tr><td>I</td><td>5142627</td><td>A</td><td>AG</td><td>2501.97</td><td>EFF=FRAME_SHIFT(HIGH||aag/aaGg|K74K?||SPAC29A4.03c.1:exon:1|||transcript:SPAC29A4.03c.1|1|1),SPLICE_SITE_REGION(LOW||||SPAC29A4.03c.1:exon:1|||transcript:SPAC29A4.03c.1|1|1)</td></tr>
<tr><td>I</td><td>5148414</td><td>TA</td><td>T</td><td>1655.97</td><td>EFF=INTERGENIC(MODIFIER||||||||||1)</td></tr>
<tr><td>I</td><td>5173087</td><td>C</td><td>CT</td><td>409.97</td><td>EFF=FRAME_SHIFT(HIGH||agc/aAgc|S132K?||SPNCRNA.1055.1:exon:1|||transcript:SPNCRNA.1055.1|1|1|WARNING_TRANSCRIPT_INCOMPLETE)</td></tr>
<tr><td>I</td><td>5368262</td><td>TC</td><td>T</td><td>1709.97</td><td>EFF=FRAME_SHIFT(HIGH||-/-|-156||SPAC4D7.09.1:exon:1|||transcript:SPAC4D7.09.1|3|1)</td></tr>
<tr><td>I</td><td>5368273</td><td>G</td><td>GT</td><td>1731.97</td><td>EFF=FRAME_SHIFT(HIGH||ggt/ggTt|G159G?||SPAC4D7.09.1:exon:1|||transcript:SPAC4D7.09.1|3|1)</td></tr>
<tr><td>I</td><td>5413497</td><td>A</td><td>AT</td><td>801.97</td><td>EFF=INTERGENIC(MODIFIER||||||||||1)</td></tr>
<tr><td>I</td><td>5426622</td><td>C</td><td>CT</td><td>78.97</td><td>EFF=FRAME_SHIFT(HIGH||aag/aaAg|K120K?||SPNCRNA.1082.1:exon:1|||transcript:SPNCRNA.1082.1|1|1|WARNING_TRANSCRIPT_MULTIPLE_STOP_CODONS)</td></tr>
<tr><td>II</td><td>146079</td><td>TC</td><td>T</td><td>1745.97</td><td>EFF=FRAME_SHIFT(HIGH||-/-|-910||SPNCRNA.1316.1:exon:1|||transcript:SPNCRNA.1316.1|1|1|WARNING_TRANSCRIPT_INCOMPLETE)</td></tr>
</table>

Indels

<table>
<tr><th>#CHROM</th><th>POS</th><th>REF</th><th>ALT</th><th>QUAL</th><th></th></tr>
<tr><td>II</td><td>295849</td><td>CT</td><td>C</td><td>908.97</td><td>EFF=FRAME_SHIFT(HIGH||-/-|-374||SPNCRNA.1336.1:exon:1|||transcript:SPNCRNA.1336.1|1|1|WARNING_TRANSCRIPT_MULTIPLE_STOP_CODONS)</td></tr>
<tr><td>II</td><td>528051</td><td>A</td><td>ATGTGTGTGTGTG</td><td>0.0</td><td>EFF=CODON_INSERTION(MODERATE||ata/aCACACACACACAta|I85THTHI||SPNCRNA.134.1:exon:1|||transcript:SPNCRNA.134.1|1|1|WARNING_TRANSCRIPT_INCOMPLETE),CODON_INSERTION(MODERATE||atg/aTGTGTGTGTGTGtg|M337MCVCV||SPNCRNA.133.1:exon:1|||transcript:SPNCRNA.133.1|1|1|WARNING_TRANSCRIPT_INCOMPLETE),UTR_3_PRIME(MODIFIER||1410|||SPBC1685.13.1:exon:1|||transcript:SPBC1685.13.1|1|1)</td></tr>
<tr><td>II</td><td>602525</td><td>TA</td><td>T</td><td>332.97</td><td>EFF=INTERGENIC(MODIFIER||||||||||1)</td></tr>
<tr><td>II</td><td>603279</td><td>AT</td><td>A</td><td>1399.97</td><td>EFF=FRAME_SHIFT(HIGH||-/-|-114||SPNCRNA.317.1:exon:1|||transcript:SPNCRNA.317.1|1|1|WARNING_TRANSCRIPT_INCOMPLETE)</td></tr>
<tr><td>II</td><td>603305</td><td>AC</td><td>A</td><td>1719.97</td><td>EFF=FRAME_SHIFT(HIGH||-/-|-123||SPNCRNA.317.1:exon:1|||transcript:SPNCRNA.317.1|1|1|WARNING_TRANSCRIPT_INCOMPLETE)</td></tr>
<tr><td>II</td><td>700009</td><td>TA</td><td>T</td><td>1913.97</td><td>EFF=INTERGENIC(MODIFIER||||||||||1)</td></tr>
<tr><td>II</td><td>888599</td><td>AC</td><td>A</td><td>2379.97</td><td>EFF=UTR_5_PRIME(MODIFIER||58|||SPBC713.11c.1:exon:1|||transcript:SPBC713.11c.1|1|1)</td></tr>
<tr><td>II</td><td>903106</td><td>G</td><td>GT</td><td>2313.97</td><td>EFF=UTR_3_PRIME(MODIFIER||49|||SPBC216.04c.1:exon:1|||transcript:SPBC216.04c.1|1|1)</td></tr>
<tr><td>II</td><td>1079843</td><td>T</td><td>TA</td><td>81.97</td><td>EFF=INTERGENIC(MODIFIER||||||||||1)</td></tr>
<tr><td>II</td><td>1146497</td><td>A</td><td>AT</td><td>0.0</td><td>EFF=UTR_5_PRIME(MODIFIER||714|||wis1:exon:1|||transcript:wis1|1|1)</td></tr>
<tr><td>II</td><td>1163154</td><td>AT</td><td>A</td><td>2778.97</td><td>EFF=UTR_5_PRIME(MODIFIER||73|||SPBC409.12c.1:exon:1|||transcript:SPBC409.12c.1|1|1)</td></tr>
<tr><td>II</td><td>1308618</td><td>GT</td><td>G</td><td>988.97</td><td>EFF=INTERGENIC(MODIFIER||||||||||1)</td></tr>
<tr><td>II</td><td>1555919</td><td>A</td><td>ATTTATT</td><td>705.97</td><td>EFF=CODON_INSERTION(MODERATE||ttt/TTTATTttt|F360FIF||SPNCRNA.1443.1:exon:1|||transcript:SPNCRNA.1443.1|1|1|WARNING_TRANSCRIPT_MULTIPLE_STOP_CODONS)</td></tr>
<tr><td>II</td><td>1641053</td><td>T</td><td>TCTAGTGTCAGTA</td><td>526.97</td><td>EFF=CODON_INSERTION(MODERATE||tct/tCTAGTGTCAGTAct|S314SSVST||SPNCRNA.373.1:exon:1|||transcript:SPNCRNA.373.1|1|1|WARNING_TRANSCRIPT_INCOMPLETE)</td></tr>
<tr><td>II</td><td>1678205</td><td>TA</td><td>T</td><td>236.97</td><td>EFF=UTR_5_PRIME(MODIFIER||630|||SPBC19C2.04c.1:exon:1|||transcript:SPBC19C2.04c.1|1|1)</td></tr>
<tr><td>II</td><td>1716287</td><td>TG</td><td>T</td><td>352.97</td><td>EFF=INTERGENIC(MODIFIER||||||||||1)</td></tr>
<tr><td>II</td><td>1747580</td><td>TA</td><td>T</td><td>990.97</td><td>EFF=UTR_5_PRIME(MODIFIER||155|||SPBC1D7.03.1:exon:1|||transcript:SPBC1D7.03.1|1|1)</td></tr>
<tr><td>II</td><td>1816760</td><td>T</td><td>TC</td><td>0.0</td><td>EFF=UTR_5_PRIME(MODIFIER||12|||SPBC9B6.02c.1:exon:1|||transcript:SPBC9B6.02c.1|1|1)</td></tr>
<tr><td>II</td><td>1818220</td><td>CTCTCT</td><td>C</td><td>3007.97</td><td>EFF=UTR_5_PRIME(MODIFIER||52|||SPBC9B6.03.1:exon:1|||transcript:SPBC9B6.03.1|1|1)</td></tr>
<tr><td>II</td><td>1861420</td><td>TG</td><td>T</td><td>2919.97</td><td>EFF=INTERGENIC(MODIFIER||||||||||1)</td></tr>
<tr><td>II</td><td>1867795</td><td>A</td><td>AT</td><td>240.97</td><td>EFF=INTERGENIC(MODIFIER||||||||||1)</td></tr>
<tr><td>II</td><td>1873779</td><td>T</td><td>TA</td><td>1500.97</td><td>EFF=UTR_5_PRIME(MODIFIER||679|||SPBC3H7.05c.1:exon:1|||transcript:SPBC3H7.05c.1|1|1)</td></tr>
<tr><td>II</td><td>1877294</td><td>A</td><td>AT</td><td>309.97</td><td>EFF=FRAME_SHIFT(HIGH||ttt/Tttt|F1F?||SPNCRNA.1472.1:exon:1|||transcript:SPNCRNA.1472.1|1|1|WARNING_TRANSCRIPT_MULTIPLE_STOP_CODONS)</td></tr>
<tr><td>II</td><td>1941966</td><td>A</td><td>AT</td><td>778.97</td><td>EFF=FRAME_SHIFT(HIGH||ctc/cAtc|L142H?||SPNCRNA.1479.1:exon:1|||transcript:SPNCRNA.1479.1|1|1|WARNING_TRANSCRIPT_INCOMPLETE)</td></tr>
<tr><td>II</td><td>1941982</td><td>A</td><td>AT</td><td>732.97</td><td>EFF=FRAME_SHIFT(HIGH||ctc/ctcA|L136L?||SPNCRNA.1479.1:exon:1|||transcript:SPNCRNA.1479.1|1|1|WARNING_TRANSCRIPT_INCOMPLETE)</td></tr>
<tr><td>II</td><td>1948953</td><td>GA</td><td>G</td><td>548.97</td><td>EFF=INTRON(MODIFIER|||||SPBC16E9.16c.1:exon:1|||transcript:SPBC16E9.16c.1|2|1),SPLICE_SITE_ACCEPTOR(HIGH|||||SPBC16E9.16c.1:exon:1|||transcript:SPBC16E9.16c.1|3|1),SPLICE_SITE_DONOR(HIGH|||||SPBC16E9.16c.1:exon:1|||transcript:SPBC16E9.16c.1|2|1)</td></tr>
<tr><td>II</td><td>1950050</td><td>A</td><td>AG</td><td>769.97</td><td>EFF=INTRON(MODIFIER|||||SPBC16E9.16c.1:exon:1|||transcript:SPBC16E9.16c.1|1|1),SPLICE_SITE_ACCEPTOR(HIGH|||||SPBC16E9.16c.1:exon:1|||transcript:SPBC16E9.16c.1|2|1),SPLICE_SITE_DONOR(HIGH|||||SPBC16E9.16c.1:exon:1|||transcript:SPBC16E9.16c.1|1|1)</td></tr>
<tr><td>II</td><td>1955495</td><td>T</td><td>TA</td><td>0.0</td><td>EFF=FRAME_SHIFT(HIGH||tta/ttAa|L910L?||SPNCRNA.1481.1:exon:1|||transcript:SPNCRNA.1481.1|1|1|WARNING_TRANSCRIPT_INCOMPLETE)</td></tr>
<tr><td>II</td><td>1960392</td><td>A</td><td>AG</td><td>2908.97</td><td>EFF=FRAME_SHIFT(HIGH||gaa/gaaC|E475E?||SPBC1E8.03c.1:exon:1|||transcript:SPBC1E8.03c.1|1|1),INTRON(MODIFIER|||||SPBC1E8.02.1:exon:1|||transcript:SPBC1E8.02.1|1|1)</td></tr>
<tr><td>II</td><td>1970829</td><td>C</td><td>CATCT</td><td>3040.97</td><td>EFF=UTR_5_PRIME(MODIFIER||84|||SPBC1E8.05.1:exon:1|||transcript:SPBC1E8.05.1|1|1)</td></tr>
<tr><td>II</td><td>1987101</td><td>CG</td><td>C</td><td>2882.97</td><td>EFF=FRAME_SHIFT(HIGH||-/-|-376||SPBC1A4.06c.1:exon:1|||transcript:SPBC1A4.06c.1|3|1),SPLICE_SITE_REGION(LOW|||||SPBC1A4.06c.1:exon:1|||transcript:SPBC1A4.06c.1|3|1),UTR_3_PRIME(MODIFIER||431|||SPBC1A4.05.1:exon:1|||transcript:SPBC1A4.05.1|2|1)</td></tr>
<tr><td>II</td><td>1987117</td><td>TG</td><td>T</td><td>2754.97</td><td>EFF=FRAME_SHIFT(HIGH||-/-|-370||SPBC1A4.06c.1:exon:1|||transcript:SPBC1A4.06c.1|3|1),UTR_3_PRIME(MODIFIER||447|||SPBC1A4.05.1:exon:1|||transcript:SPBC1A4.05.1|2|1)</td></tr>
<tr><td>II</td><td>2049891</td><td>AT</td><td>A</td><td>1705.97</td><td>EFF=FRAME_SHIFT(HIGH||-/-|-555||SPBC29A3.06.1:exon:1|||transcript:SPBC29A3.06.1|1|1),SPLICE_SITE_REGION(LOW|||||SPBC29A3.06.1:exon:1|||transcript:SPBC29A3.06.1|1|1)</td></tr>
<tr><td>II</td><td>2053516</td><td>G</td><td>GC</td><td>2418.97</td><td>EFF=FRAME_SHIFT(HIGH||gcc/gCcc|A162A?||SPBC29A3.08.1:exon:1|||transcript:SPBC29A3.08.1|1|1),SPLICE_SITE_REGION(LOW|||||SPBC29A3.08.1:exon:1|||transcript:SPBC29A3.08.1|1|1)</td></tr>
<tr><td>II</td><td>2108180</td><td>T</td><td>TA</td><td>3930.97</td><td>EFF=FRAME_SHIFT(HIGH||tgg/tggT|W202W?||SPBC23G7.06c.1:exon:1|||transcript:SPBC23G7.06c.1|1|1),SPLICE_SITE_REGION(LOW|||||SPBC23G7.06c.1:exon:1|||transcript:SPBC23G7.06c.1|1|1)</td></tr>
<tr><td>II</td><td>2147726</td><td>G</td><td>GT</td><td>1700.97</td><td>EFF=UTR_3_PRIME(MODIFIER||29|||SPBC1711.07.1:exon:1|||transcript:SPBC1711.07.1|1|1)</td></tr>
<tr><td>II</td><td>2187407</td><td>G</td><td>GA</td><td>1813.97</td><td>EFF=INTERGENIC(MODIFIER||||||||||1)</td></tr>
<tr><td>II</td><td>2199987</td><td>ATAACC</td><td>A</td><td>1214.97</td><td>EFF=INTERGENIC(MODIFIER||||||||||1)</td></tr>
<tr><td>II</td><td>2219928</td><td>A</td><td>AT</td><td>2544.97</td><td>EFF=FRAME_SHIFT(HIGH||cat/caAt|H131Q?||SPBC14C8.09c.1:exon:1|||transcript:SPBC14C8.09c.1|2|1),UTR_5_PRIME(MODIFIER||834|||SPBC14C8.10.1:five_prime_UTR:1|||transcript:SPBC14C8.10.1|2|1)</td></tr>
<tr><td>II</td><td>2293297</td><td>GA</td><td>G</td><td>2079.97</td><td>EFF=UTR_3_PRIME(MODIFIER||95|||SPBC16H5.04.1:exon:1|||transcript:SPBC16H5.04.1|3|1)</td></tr>
<tr><td>II</td><td>2322353</td><td>G</td><td>GA</td><td>1110.97</td><td>EFF=UTR_3_PRIME(MODIFIER||22|||SPBC24C6.02.1:exon:1|||transcript:SPBC24C6.02.1|2|1)</td></tr>
<tr><td>II</td><td>2592423</td><td>T</td><td>TA</td><td>449.97</td><td>EFF=INTRON(MODIFIER|||||SPBC2G5.07c.1:exon:1|||transcript:SPBC2G5.07c.1|3|1)</td></tr>
<tr><td>II</td><td>2630607</td><td>C</td><td>CA</td><td>0.0</td><td>EFF=UTR_3_PRIME(MODIFIER||153|||SPBC6B1.02.1:exon:1|||transcript:SPBC6B1.02.1|1|1)</td></tr>
<tr><td>II</td><td>2709414</td><td>G</td><td>GC</td><td>1997.97</td><td>EFF=FRAME_SHIFT(HIGH||gct/gCct|A447A?||SPBC4F6.10.1:exon:1|||transcript:SPBC4F6.10.1|1|1),SPLICE_SITE_REGION(LOW|||||SPBC4F6.10.1:exon:1|||transcript:SPBC4F6.10.1|1|1)</td></tr>
<tr><td>II</td><td>2713912</td><td>C</td><td>CT</td><td>383.97</td><td>EFF=UTR_3_PRIME(MODIFIER||29|||SPBC4F6.12.1:exon:1|||transcript:SPBC4F6.12.1|2|1)</td></tr>
<tr><td>II</td><td>2755338</td><td>CAT</td><td>C</td><td>309.97</td><td>EFF=INTERGENIC(MODIFIER||||||||||1)</td></tr>
<tr><td>II</td><td>2788933</td><td>C</td><td>CG</td><td>3400.97</td><td>EFF=UTR_3_PRIME(MODIFIER||137|||SPBC32F12.03c.1:exon:1|||transcript:SPBC32F12.03c.1|1|1)</td></tr>
<tr><td>II</td><td>2798040</td><td>CT</td><td>C</td><td>2394.97</td><td>EFF=FRAME_SHIFT(HIGH||-/-|-156||SPBC32F12.08c.1:exon:1|||transcript:SPBC32F12.08c.1|1|1)</td></tr>
<tr><td>II</td><td>2798514</td><td>AG</td><td>A</td><td>1714.97</td><td>EFF=UTR_5_PRIME(MODIFIER||8|||SPBC32F12.08c.1:exon:1|||transcript:SPBC32F12.08c.1|1|1)</td></tr>
<tr><td>II</td><td>2811495</td><td>CA</td><td>C</td><td>3589.97</td><td>EFF=INTRON(MODIFIER|||||SPBC32F12.12c.1:exon:1|||transcript:SPBC32F12.12c.1|2|1)</td></tr>
<tr><td>II</td><td>2938956</td><td>C</td><td>CT</td><td>103.97</td><td>EFF=INTERGENIC(MODIFIER||||||||||1)</td></tr>
<tr><td>II</td><td>3040332</td><td>C</td><td>CG</td><td>2719.97</td><td>EFF=INTRON(MODIFIER|||||SPBC13E7.01.1:exon:1|||transcript:SPBC13E7.01.1|1|1),SPLICE_SITE_ACCEPTOR(HIGH|||||SPBC13E7.01.1:exon:1|||transcript:SPBC13E7.01.1|2|1),SPLICE_SITE_DONOR(HIGH|||||SPBC13E7.01.1:exon:1|||transcript:SPBC13E7.01.1|1|1)</td></tr>
<tr><td>II</td><td>3058544</td><td>TG</td><td>T</td><td>165.97</td><td>EFF=UTR_3_PRIME(MODIFIER||2089|||SPBC13E7.09.1:exon:1|||transcript:SPBC13E7.09.1|4|1),UTR_5_PRIME(MODIFIER||89|||SPBC13E7.10c.1:exon:1|||transcript:SPBC13E7.10c.1|1|1)</td></tr>
<tr><td>II</td><td>3619003</td><td>A</td><td>AG</td><td>1997.97</td><td>EFF=FRAME_SHIFT(HIGH||agt/aGgt|S240R?||SPBC16D10.10.1:exon:1|||transcript:SPBC16D10.10.1|5|1)</td></tr>
<tr><td>II</td><td>3838101</td><td>CA</td><td>C</td><td>92.97</td><td>EFF=INTERGENIC(MODIFIER||||||||||1)</td></tr>
</table>

<table>
<tr><th colspan="6">Indels</th></tr>
<tr><th>#CHROM</th><th>POS</th><th>REF</th><th>ALT</th><th>QUAL</th><th></th></tr>
<tr><td>II</td><td>3860185</td><td>CTAGTA</td><td>C</td><td>1620.97</td><td>EFF=INTERGENIC(MODIFIER||||||||||1)</td></tr>
<tr><td>II</td><td>4254960</td><td>GA</td><td>G</td><td>1694.97</td><td>EFF=UTR_3_PRIME(MODIFIER||521|||SPBC1652.01.1:exon:1|||transcript:SPBC1652.01.1|1|1)</td></tr>
<tr><td>II</td><td>4308414</td><td>C</td><td>CA</td><td>85.97</td><td>EFF=INTRON(MODIFIER|||||SPBC543.05c.1:exon:1|||transcript:SPBC543.05c.1|4|1)</td></tr>
<tr><td>II</td><td>4537387</td><td>AAATAAGAG</td><td>A</td><td>2328.97</td><td>SF=0,1</td></tr>
<tr><td>III</td><td>138018</td><td>C</td><td>CA</td><td>554.97</td><td>EFF=UTR_3_PRIME(MODIFIER||77|||SPCC330.14c.1:exon:1|||transcript:SPCC330.14c.1|1|1)</td></tr>
<tr><td>III</td><td>140890</td><td>C</td><td>CT</td><td>1105.97</td><td>EFF=UTR_5_PRIME(MODIFIER||698|||SPCC320.14.1:exon:1|||transcript:SPCC320.14.1|1|1)</td></tr>
<tr><td>III</td><td>221285</td><td>TTGCATTTCCTATCCCTCCAAA</td><td>T</td><td>1103.97</td><td>EFF=CODON_DELETION(MODERATE||tttggagggataggaaatgca/-|FGGIGNA329-||SPCC548.03c.1:exon:1|||transcript:SPCC548.03c.1|6|1)</td></tr>
<tr><td>III</td><td>719955</td><td>AC</td><td>A</td><td>979.97</td><td>EFF=INTERGENIC(MODIFIER||||||||||1)</td></tr>
<tr><td>III</td><td>720683</td><td>A</td><td>AT</td><td>40.97</td><td>EFF=FRAME_SHIFT(HIGH||aaa/aaaA|K295K?||SPNCRNA.1154.1:exon:1|||transcript:SPNCRNA.1154.1|1|1|WARNING_TRANSCRIPT_MULTIPLE_STOP_CODONS)</td></tr>
<tr><td>III</td><td>750204</td><td>CG</td><td>C</td><td>0.0</td><td>EFF=FRAME_SHIFT(HIGH||-/-|-312||SPCC14G10.04.1:exon:1|||transcript:SPCC14G10.04.1|4|1)</td></tr>
<tr><td>III</td><td>867865</td><td>CT</td><td>C</td><td>237.97</td><td>EFF=UTR_5_PRIME(MODIFIER||130|||SPCC16A11.01.1:exon:1|||transcript:SPCC16A11.01.1|1|1)</td></tr>
<tr><td>III</td><td>903387</td><td>A</td><td>AAAT</td><td>2041.97</td><td>EFF=UTR_3_PRIME(MODIFIER||162|||SPCC24B10.02c.1:exon:1|||transcript:SPCC24B10.02c.1|1|1),UTR_3_PRIME(MODIFIER||251|||SPCC16A11.17.1:exon:1|||transcript:SPCC16A11.17.1|1|1)</td></tr>
<tr><td>III</td><td>1016311</td><td>G</td><td>GC</td><td>2368.97</td><td>EFF=INTERGENIC(MODIFIER||||||||||1)</td></tr>
<tr><td>III</td><td>1079346</td><td>A</td><td>AG</td><td>163.97</td><td>EFF=INTERGENIC(MODIFIER||||||||||1)</td></tr>
<tr><td>III</td><td>1168405</td><td>TA</td><td>T</td><td>567.97</td><td>EFF=INTERGENIC(MODIFIER||||||||||1)</td></tr>
<tr><td>III</td><td>1663670</td><td>A</td><td>AT</td><td>0.0</td><td>EFF=FRAME_SHIFT(HIGH||gta/gAta|V317D?||SPCC1902.02.1:exon:1|||transcript:SPCC1902.02.1|3|1)</td></tr>
<tr><td>III</td><td>1663677</td><td>GCTCCTTGGCGATTTC</td><td>G</td><td>0.0</td><td>EFF=CODON_DELETION(MODERATE||gaaatcgccaaggag/-|EIAKE310-||SPCC1902.02.1:exon:1|||transcript:SPCC1902.02.1|3|1)</td></tr>
<tr><td>III</td><td>1663694</td><td>ACTG</td><td>A</td><td>0.0</td><td>EFF=CODON_CHANGE_PLUS_CODON_DELETION(MODERATE||ccagtc/ctc|PV308L||SPCC1902.02.1:exon:1|||transcript:SPCC1902.02.1|3|1)</td></tr>
<tr><td>III</td><td>1685487</td><td>G</td><td>GA</td><td>2207.97</td><td>EFF=INTERGENIC(MODIFIER||||||||||1)</td></tr>
<tr><td>III</td><td>1756400</td><td>TA</td><td>T</td><td>3541.97</td><td>EFF=INTRON(MODIFIER|||||SPCC1450.12.1:exon:1|||transcript:SPCC1450.12.1|4|1)</td></tr>
<tr><td>III</td><td>1774235</td><td>T</td><td>TGATC</td><td>6019.97</td><td>EFF=FRAME_SHIFT(HIGH||aag/aagGATC|K398KD?||SPCC1442.04c.1:exon:1|||transcript:SPCC1442.04c.1|1|1),SPLICE_SITE_REGION(LOW|||||SPCC1442.04c.1:exon:1|||transcript:SPCC1442.04c.1|1|1)</td></tr>
<tr><td>III</td><td>1929127</td><td>AG</td><td>A</td><td>152.97</td><td>EFF=INTERGENIC(MODIFIER||||||||||1)</td></tr>
<tr><td>III</td><td>2122889</td><td>T</td><td>TA</td><td>1612.97</td><td>EFF=FRAME_SHIFT(HIGH||tta/ttTa|L299F?||SPCC126.04c.1:exon:1|||transcript:SPCC126.04c.1|2|1)</td></tr>
<tr><td>III</td><td>2352293</td><td>A</td><td>AG</td><td>0.0</td><td>EFF=UTR_5_PRIME(MODIFIER||88|||SPCC70.03c.1:exon:1|||transcript:SPCC70.03c.1|1|1)</td></tr>
</table>

Supplementary Table 1. Differences between PB1623 and 972 h- established on 243 sequenced genomes. SNVs and indels that are shared among the 243 strains previously sequenced and polymorphic to the 972 h^- reference strain are listed. The predicted effect of the polymorphisms was obtained with the snpEff algorithm [1]. The sequencing data are publicly available at the NCBI BioProject database (accession no: PRJNA413662).

Supplementary Table 2: Phenotypic characterization of the 150 long-term survivors.

Clone #	Sorbitol 2 M	KCl 0.8 M	SDS 0.01%	TBZ 14 µg/mL	HU 4 mM	18 °C	37 °C	CaCl2 0.3 M	Strain name	S/MAPK mutation
Wild±type	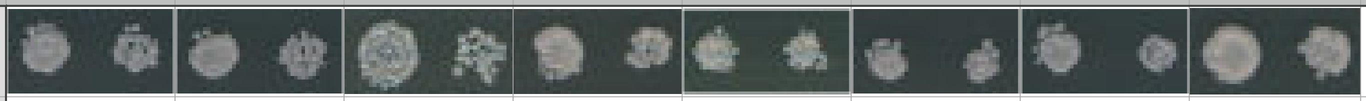								PB1623	
1	+	+	+	+	±	+	+			
2	+	+	+	+	+	+	+			
3	+	+	+	+	±	+	+			
4	+	+	+	+	±	+	+			
5	+	+	+	+	±	+	+			
6	+	+	+	+	+	+	+			
7	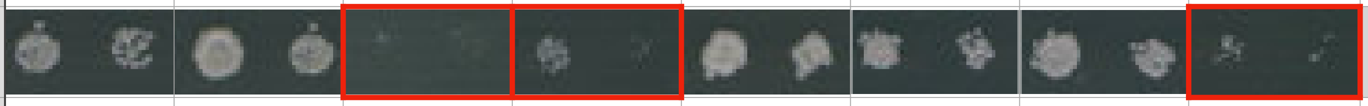								1P	*pmc1*-T326A
8	+	+	+	+	±	+	+			
9	+	+	+	+	±	+	+			
10	+	+	+	+	±	+	+			
11	+	+	+	+	±	+	+			
12	+	+	+	+	+	+	±			
13	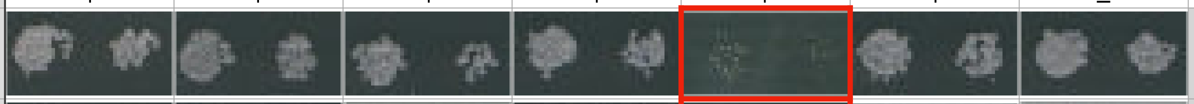							ND		
14	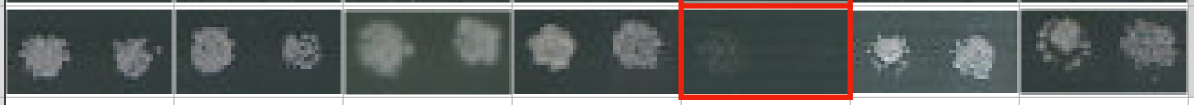							ND		
15	+	+	+	±	+	+	+			
16	+	+	+	+	+	+	+			
17	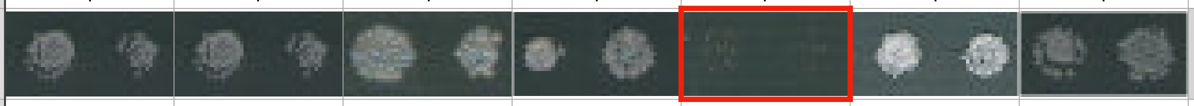							ND		
18	+	+	+	+	±	+	+			
19	+	+	+	+	±	+	+			
20	+	+	+	+	±	+	+			
21	+	+	+	+	±	+	+			
22	+	+	+	+	±	+	+			
23	+	+	+	±	+	+	+			
24	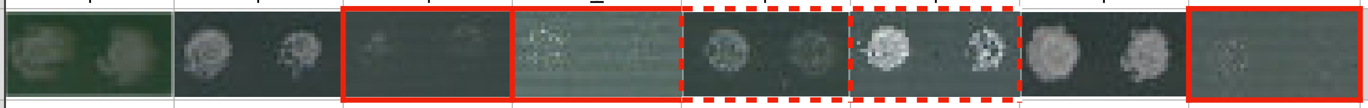								2P	*pmc1*-2906 -C
25	+	+	+	+	±	+	+			
26	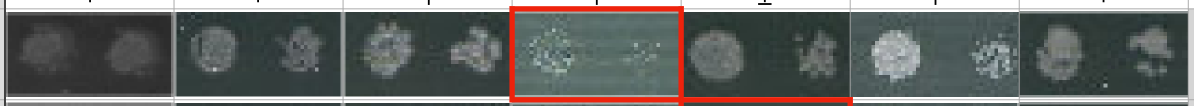							ND		
27	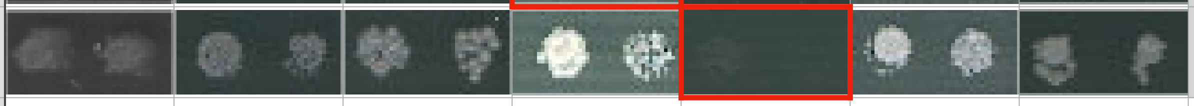							ND		
28	+	+	+	+	+	+	+			
29	+	+	+	+	±	+	+			
30	+	+	+	+	±	+	+			
31	+	+	+	+	±	+	+			
32	+	+	+	+	±	+	+			
33	+	+	+	+	±	+	+			
34	+	+	+	+	±	+	+			
35	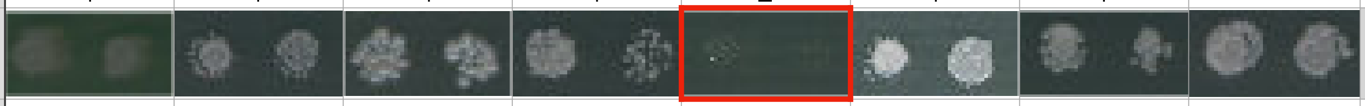								3P	*sgf73*-896 +T
36	+	+	+	+	±	+	+			
37	+	+	+	+	+	+	+			
38	+	+	+	+	±	+	+			
39	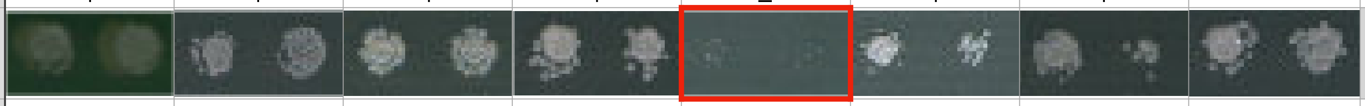								4P	*sgf73*-896 +T
40	+	+	+	+	+	+	±			
41	+	+	+	+	±	+	+			
42	+	+	+	+	+	+	+			
43	+	+	+	+	±	+	+			
44	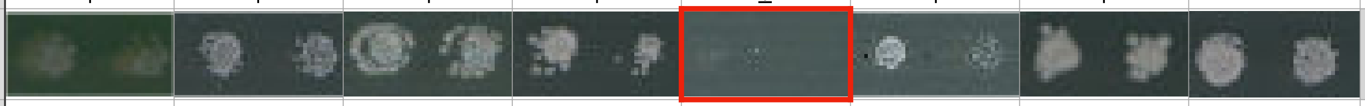								5P	*sgf73*-896 +T
45	+	+	+	+	±	+	+			
46	+	+	+	+	±	+	+			
47	+	+	+	+	+	+	+			
48	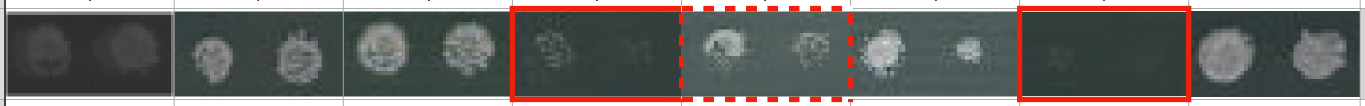								6P	*sty1*-C74G
49	+	+	+	±	±	+	+			
50	+	+	+	+	±	+	+			
51	+	+	+	+	±	+	+			
52	+	+	+	+	±	+	+			
53	+	+	+	+	+	+	+			
54	+	+	+	+	±	+	+			

Strong phenotype

Sensitivity

Mild sensitivity

ND: Not Determined

Clone #	Sorbitol 2 M	KCl 0.8 M	SDS 0.01%	TBZ 14 µg/mL	HU 4 mM	18 °C	37 °C	CaCl2 0.3 M	Strain name	S/MAPK mutation
55	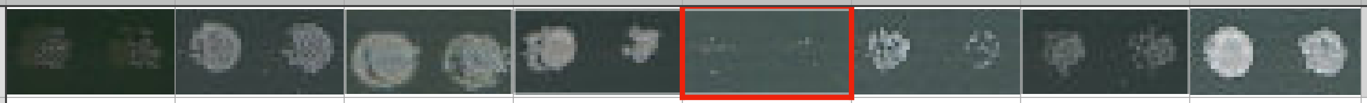								7P	*sgf73*-896 +T
56	+	+	+	+	±	+	+			
57	+	+	+	+	±	+	+			
58	+	+	+	+	+	+	+			
59	+	+	+	+	+	+	+			
60	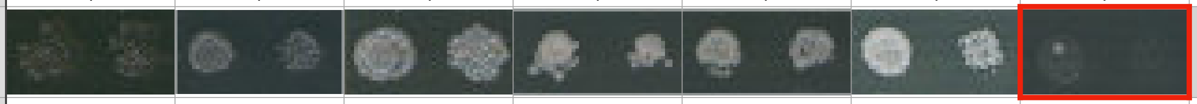							ND		
61	+	+	+	+	+	+	+			
62	+	+	+	+	+	+	+			
63	+	+	+	+	+	+	+			
64	+	+	+	+	+	+	+			
65	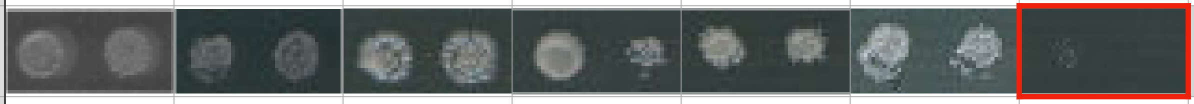							ND		
66	+	+	+	+	+	+	+			
67	+	+	+	+	±	+	+			
68	+	+	+	+	+	+	+			
69	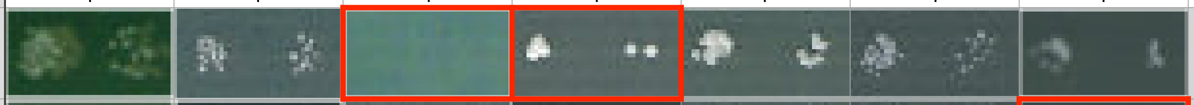							–	8P	*pmc1*-C382T
70	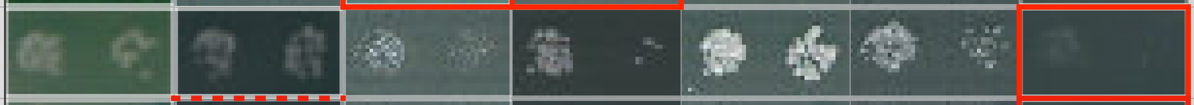							ND		
71	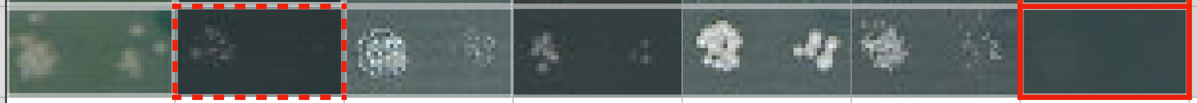							+	9P	*win1*-1086 -G
72	+	+	+	+	+	+	+			
73	+	+	+	+	+	+	+			
74	+	+	+	+	+	+	+			
75	+	+	+	+	+	+	+			
76	+	+	+	+	+	+	+			
77	+	+	+	+	+	+	+			
78	+	+	+	+	+	+	+			
79	+	+	+	+	+	+	+			
80	+	+	+	+	+	+	+			
81	+	+	+	+	+	+	+			
82	+	+	+	+	+	+	+			
83	+	+	+	+	+	+	+			
84	+	+	+	+	+	+	+			
85	+	+	+	+	+	+	+			
86	+	+	+	+	+	+	+			
87	+	+	+	+	+	+	+			
88	+	+	+	+	+	+	+			
89	+	+	+	+	+	+	+			
90	+	+	+	+	+	+	+			
91	+	+	+	+	+	+	+			
92	+	+	+	+	+	+	+			
93	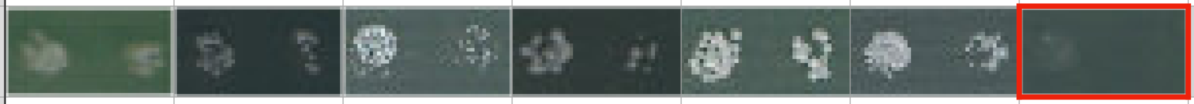							ND	10P	*cut1 + qcr6*
94	+	+	+	+	+	+	+			
95	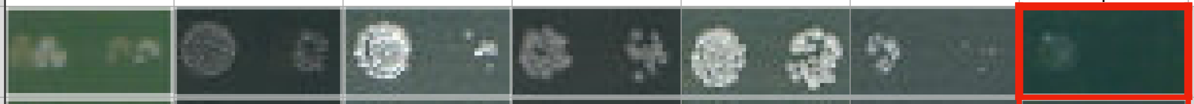							ND		
96	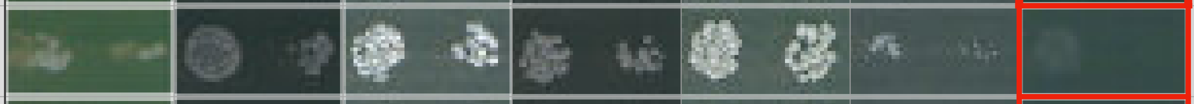							ND		
97	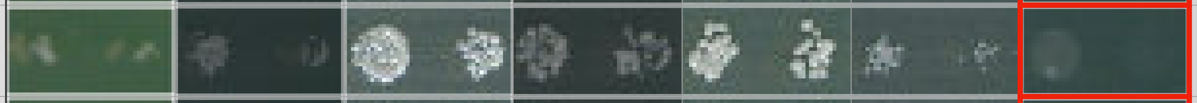							ND		
98	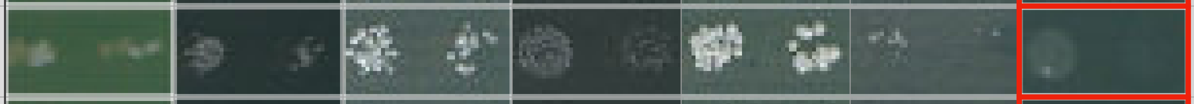							ND	11P	SPCC14G10.04
99	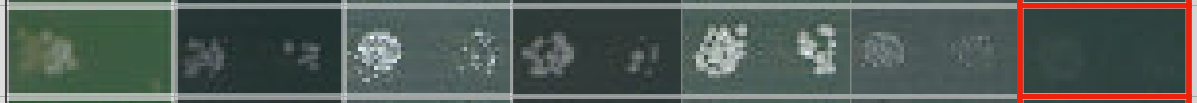							ND		
100	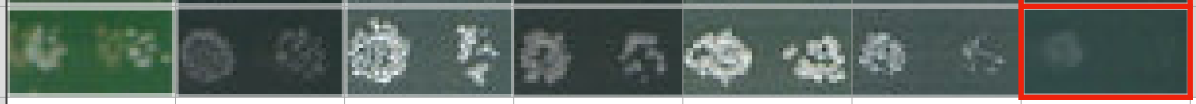							ND		
101	+	+	+	+	+	+	+			
102	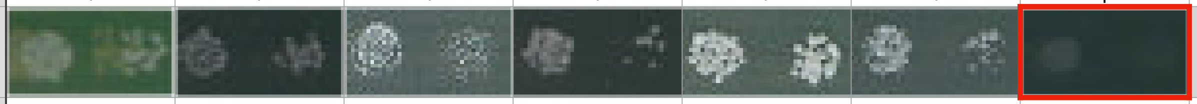							ND		
103	+	+	+	+	+	+	+			
104	+	+	+	+	+	+	+			
105	+	+	+	+	+	+	+			
106	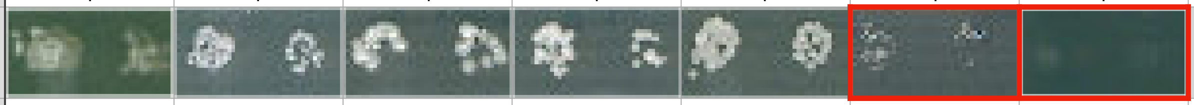							ND	12P	*meu23*
107	+	+	+	+	+	+	+			
108	+	+	+	+	+	+	+			

Clone #	Sorbitol 2 M	KCl 0.8 M	SDS 0.01%	TBZ 14 µg/mL	HU 4 mM	18 °C	37 °C	CaCl2 0.3 M	Strain name	S/MAPK mutation
109	+	+	+	+	+	+	+			
110	+	+	+	+	+	+	+			
111	+	+	+	+	+	+	+			
112	+	+	+	+	+	+	+			
113	+	+	+	+	+	+	±			
114	+	+	+	+	+	+	+			
115	+	+	+	+	+	+	+			
116	+	+	+	+	+	+	+			
117	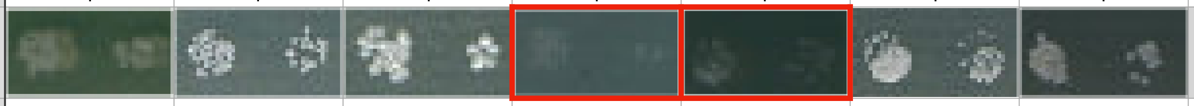							ND		
118	+	+	+	+	+	+	+			
119	+	+	+	+	+	+	+			
120	+	+	+	+	+	+	+			
121	+	+	+	+	+	+	+			
122	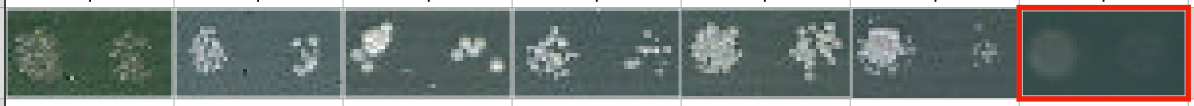							ND		
123	+	+	+	+	+	+	+			
124	+	+	+	+	+	+	+			
125	+	+	+	+	+	+	+			
126	+	+	+	+	+	+	+			
127	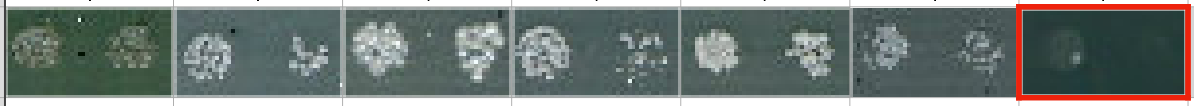							ND		
128	+	+	+	+	+	+	±			
129	+	+	+	+	+	+	+			
130	+	+	+	+	+	+	+			
131	+	+	+	+	+	+	+			
132	+	+	+	+	+	+	+			
133	+	+	+	+	+	+	+			
134	+	+	+	+	+	+	+			
135	+	+	+	+	+	+	+			
136	+	+	+	+	+	+	+			
137	+	±	+	+	+	+	±			
138	+	+	+	+	+	+	+			
139	+	+	+	+	+	+	+			
140	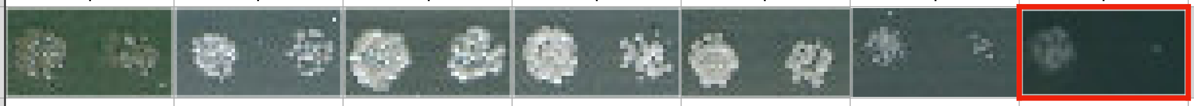							ND		
141	+	+	+	+	+	+	+			
142	+	+	+	+	+	+	±			
143	+	+	+	+	+	+	+			
144	+	+	+	+	+	+	+			
145	+	+	+	+	+	+	±			
146	+	+	+	+	+	+	+			
147	+	+	+	+	+	+	+			
148	+	+	+	+	+	+	+			
149	+	+	+	+	+	+	+			
150	+	+	+	+	+	+	+			

Supplementary Table 2. Phenotypic characterization of the 150 long-term survivors. The strains with a confirmed phenotype of sensitivity are highlighted in gray and drops of five-fold serial dilutions for all the conditions tested are shown. The twelve P strains selected for their phenotype to perform WGS and the genes of the S/MAPK pathways found mutated in these strains are also indicated.

Supplementary Table 3: Mutations found after 3 months of quiescence in the 48 genomes that were sequenced.

Strain	Chromosome	Position	Reference	Alternative	Mutation type	Gene	Allele
1R	II	945943	A	T	SNP	*gap1*	*gap1*- T1460A
2R	-	-	-	-	-	-	
3R	II	1532631	A	G	SNP	SPBC83.12	
4R	I	616209	G	GT	Insertion	*mkh1*	*mkh1*-640+A
	II	1048712	G	A	SNP	*dre2* (predicted)	*dre2*-C719T
5R	I	1591589	T	A	SNP	*thi4*	*thi4*-T1490A
6R	-	-	-	-	-	-	
7R	-	-	-	-	-	-	
8R	I	207717	C	A	SNP	*sty1*	*sty1*-G197T
	III	175767	A	ATACATCTAA	Insertion	SPCC1235.01	
9R	-	-	-	-	-	-	
10R	I	2714004	TAACCCACAA	T	Deletion	*pmc1*	*pmc1*-2920 -9 bp
11R	-	-	-	-	-	-	
12R	III	1493950	A	G	SNP	*pfl4*	*pfl4*-T818C
13R	-	-	-	-	-	-	
14R	I	255219	A	G	SNP	SPNCRNA.630 (predicted)	
	II	693417	-36bp		Deletion	SPBPJ4664.02	
	I	613911	CTATGCTTAGA	C	Deletion	*mkh1*	*mkh1-2929 -10 bp*
15R	II	4313731	T	A	SNP	*pek1*	*pek1*- T969A
	III	903386	GAAA	G	Deletion	*mcm4*	
16R	-	-	-	-	-	-	
17R	-	-	-	-	-	-	
18R	II	731112	T	TA	Insertion	*pmk1*	*pmk1*-153+A
19R	I	2715686	C	A	SNP	*pmc1*	*pmc1*-G1246T
	II	3860184	TCTAGT	T	Deletion	Intergenic	
20R	III	1493950	A	G	SNP	*pfl4*	*pfl4*-T818C
21R	II	1134749	G	T	SNP	*tif452*	*tif452*-G251T
22R	I	616209	G	GT	Insertion	*mkh1*	*mkh1*-640+A
	I	2245479	T	TCCC	Insertion	Intergenic	
23R	II	53302	G	C	SNP	Intergenic	
	I	760579		-AT	Deletion	Intergenic	
	I	801095		-AAGTATATAT	Deletion	Intergenic	
24R	I	5090834	T	TCTACTACATCCTC	Insertion	*win1*	*win1*-394 +13 bp
25R	-	-	-	-	-	-	
26R	II	1146497	C	CA	Insertion	*wis1*	- 713 +A

27R	-	-	-	-	-	-	
28R	II	4538083	GA	G	Deletion	Intergenic	
29R	I	5091713	A	ATATCCTTCACGTCGTTC	Insertion	*win1*	*win1*-1273 + 17bp
30R	I	614355	C	T	SNP	*mkh1*	*mkh1*-G2494A
	II	1649054	A	C	SNP	*rec6*	*rec6*-T533G
31R	-	-	-	-	-	-	
32R	II	1146497	C	CA	Insertion	*wis1*	- 713 +A
33R	II	4255819	T	G	SNP	SPBC1652.02-antisense-1 (predicted)	
	I	2714492	C	CAATATTATCACCAGTAAC	Insertion	*pmc1*	*pmc1*-2441+18bp
34R	II	731112	T	TA	Insertion	*pmk1*	*pmk1*-153+A
35R	II	1049917	T	TGTAA	Insertion	*dre2* (predicted)	
36R	II	1665445	C	CTTTAT	Insertion	intergenic	
1P	I	2716606	A	T	SNP	*pmc1*	*pmc1*-T326A
2P	I	2714026	AG	A	Deletion	*pmc1*	*pmc1*-2906-C
3P	III	2122890	T	TA	Insertion	*sgf73*	*sgf73-896+T*
4P	III	2122890	T	TA	Insertion	*sgf73*	*sgf73-896+T*
	I	1544016	T	A	SNP	*pabp*	*pabp*-T1152A
5P	III	2122890	T	TA	Insertion	*sgf73*	*sgf73-896+T*
6P	I	207840	G	C	SNP	*sty1*	*sty1*-G74C
	I	2499161	TG	T	Deletion	SPAC23H3.04	
	II	2437855	A	C	SNP	*map4*	*map4*-A2220C
	III	1493950	A	G	SNP	*pfl4*	*pfl4*-T818C
7P	III	2122890	T	TA	Insertion	*sgf73*	*sgf73-896+T*
8P	I	2716550	G	A	SNP	*pmc1*	*pmc1*-C382T
9P	I	5091526	TG	T	Deletion	*win1*	*win1*-1086-G
10P	III	648242	T	C	SNP	*cut1*	*cut1*-T4313C
	II	4344813	A	ATTTTCTTC	Insertion	*qcr6*	*qcr6*-T325+6 bp
11P	III	748812	A	T	SNP	SPCC14G10.04	
12P	III	748812	A	T	SNP	SPCC14G10.04	
	III	99099	A	AACTATGCAAGGGACCATGAAC	Insertion	*meu23*	*meu23*-T387 +21bp
	III	2433240	A	G	SNP	SPCC569.02c-antisense-1 (predicted)	
	III	2433242	T	A	SNP	SPCC569.02c-antisense-1 (predicted)	
	III	2433279	A	G		SPCC569.02c-antisense-1 (predicted)	
	III	2433281	A	C		SPCC569.02c-antisense-1 (predicted)	

Supplementary Table 3. Mutations found after 3 months of quiescence in the 48 genomes that were sequenced. In green are highlighted the genes belonging to the S/MAPK pathways.

**Supplementary Table 4:** Distribution of the mutants in the S/MAPK pathway among 48 strains sequenced after three months of quiescence.				
**genes**	**P strains**	**R strains**	**function**	**human homologue**
win1	1	2	SAPKKK	MAP3K4
wis1	-	2	SAPKK	MAP2K1
sty1	1	1	SAPK	p38
mkh1	-	4	MAPKKK	MEKK1
pek1	-	1	MAPKK	MEK2
pmk1	-	2	MAPK	ERK
pmc1	3	3	Calcium efflux	PMCA
sgf73	4	-	SAGA subunit	ATXN7
tif452	-	1	CAP binding	eIF4E

Supplementary Table 4. Distribution of the mutants in the S/MAPK pathways among 48 strains sequenced after three months of quiescence. The P strains were picked for their phenotype while the R strains were picked randomly.

Supplementary Table 5. Mutations identified in the targeted resequencing experiment

Sample	Mutation type	Gene	cDNA	Frequency	Ref	VarAllele	Mutation	time in quiescence	P-value < 0.05
culture_0	Insertion	*sgf73*	896	0,9 %	T	+A	+T	2_months	9,69E-23
culture_0	Insertion	*win1*	394	0,2 %	T	+CTACTACATCCTC	+CTACTACATCCTC	2_months	2,96E-02
culture_0	Insertion	*win1*	1273	0,3 %	A	+TATCCTTCACGTCGTTC	+TATCCTTCACGTCGTTC	2_months	2,44E-02
culture_0	Insertion	*mkh1*	640	13,0 %	G	+T	+A	2_months	0,00E+00
culture_0	SNP	*mkh1*	959	1,4 %	A	G	T>C	2_months	1,13E-43
culture_0	SNP	*mkh1*	1885	0,2 %	A	G	T>C	2_months	4,80E-02
culture_0	SNP	*mkh1*	2914	0,4 %	A	G	T>C	2_months	3,57E-03
culture_0	SNP	*mkh1*	3351	0,4 %	T	A	A>T	2_months	1,32E-14
culture_0	Deletion	*pmk1*	254	0,7 %	A	-T	-T	2_months	1,06E-02
culture_0	SNP	*tif452*	251	0,2 %	G	T	G>T	2_months	3,25E-05
**total = 17.7%**									
culture_0	Insertion	*sgf73*	896	4,3 %	T	+A	+T	3_months	2,12E-169
culture_0	Insertion	*win1*	394	1,5 %	T	+CTACTACATCCTC	+CTACTACATCCTC	3_months	5,40E-80
culture_0	Insertion	*win1*	549	0,3 %	T	+GCACAGAGGAC	+GCACAGAGGAC	3_months	1,66E-05
culture_0	Deletion	*win1*	756	0,2 %	C	-AG	-AG	3_months	2,20E-02
culture_0	Insertion	*win1*	1158	0,3 %	G	+TATGGTTGATGTGC	+TATGGTTGATGTGC	3_months	4,60E-02
culture_0	Insertion	*win1*	1273	1,5 %	A	+TATCCTTCACGTCGTTC	+TATCCTTCACGTCGTTC	3_months	6,51E-13
culture_0	Deletion	*win1*	1573	0,7 %	C	-A	-A	3_months	2,01E-10
culture_0	SNP	*win1*	2348	0,2 %	C	A	C>A	3_months	1,72E-03
culture_0	SNP	*win1*	2556	0,2 %	T	G	T>G	3_months	2,89E-02
culture_0	Insertion	*win1*	3010	0,9 %	T	+A	+A	3_months	7,55E-65
culture_0	SNP	*sty1*	74	0,6 %	G	C	C>G	3_months	1,12E-11
culture_0	SNP	*sty1*	197	0,5 %	C	A	G>T	3_months	6,15E-12
culture_0	Insertion	*mkh1*	640	9,0 %	G	+T	+A	3_months	6,41E-291
culture_0	SNP	*mkh1*	959	0,9 %	A	G	T>C	3_months	2,07E-29
culture_0	Insertion	*pmk1*	1091	0,3 %	C	+CAACAAACCCAACAGTCAACATCCCG	+CAACAAACCCAACAGTCAACATCCCG	3_months	2,80E-05
culture_0	SNP	*tif452*	251	0,7 %	G	T	G>T	3_months	2,11E-40
**total = 22.1%**									
culture_1	Insertion	*win1*	1273	0,3 %	A	+TATCCTTCACGTCGTTC	+TATCCTTCACGTCGTTC	2_months	1,73E-02
culture_1	Insertion	*win1*	3010	0,9 %	T	+A	+A	2_months	3,13E-79
culture_1	Insertion	*sty1*	216	0,4 %	C	+TTTAATAGT	+ACTATTAAA	2_months	5,70E-13
culture_1	SNP	*sty1*	647	1,1 %	C	G	G>C	2_months	5,47E-18
culture_1	SNP	*mkh1*	639	1,9 %	T	C	A>G	2_months	7,29E-63
culture_1	Insertion	*mkh1*	640	21,3 %	G	+T	+A	2_months	0,00E+00
culture_1	SNP	*mkh1*	2378	0,8 %	G	C	C>G	2_months	1,97E-36
culture_1	SNP	*mkh1*	3138	0,3 %	T	G	A>C	2_months	8,15E-08
culture_1	Deletion	*mkh1*	3236	1,6 %	A	-GCATTCACAGTAAAGCAT	-ATGCTTTACTGTGAATGC	2_months	1,34E-102
culture_1	SNP	*pmk1*	497	1,2 %	G	T	G>T	2_months	1,13E-27
**total = 29.8%**									
culture_1	SNP	*win1*	2666	1,2 %	C	T	C>T	3_months	3,02E-59
culture_1	Insertion	*win1*	3010	1,2 %	T	+A	+A	3_months	2,62E-56
culture_1	Insertion	*sty1*	216	1,8 %	C	+TTTAATAGT	+ACTATTAAA	3_months	6,60E-52
culture_1	Insertion	*mkh1*	640	10,2 %	G	+T	+A	3_months	4,73E-234
culture_1	Deletion	*mkh1*	3236	0,2 %	A	-GCATTCACAGTAAAGCAT	-ATGCTTTACTGTGAATGC	3_months	1,91E-03
culture_1	SNP	*pmk1*	577	6,1 %	T	G	T>G	3_months	5,94E-146
**total = 20.7%**									
culture_2	Insertion	*sgf73*	896	9,6 %	T	+A	+T	2_months	0,00E+00
culture_2	Insertion	*win1*	393	0,6 %	A	+TCTACTACATCCT	+TCTACTACATCCT	2_months	7,32E-19
culture_2	SNP	*win1*	811	0,8 %	C	A	C>A	2_months	5,50E-38
culture_2	Deletion	*win1*	2339	0,8 %	T	-ACGTA	-ACGTA	2_months	6,53E-18
culture_2	SNP	*win1*	2379	0,3 %	T	G	T>G	2_months	5,01E-10
culture_2	SNP	*sty1*	188	3,7 %	C	G	G>C	2_months	1,30E-146
culture_2	SNP	*mkh1*	250	4,7 %	C	A	G>T	2_months	2,41E-227
culture_2	Insertion	*mkh1*	640	27,0 %	G	+T	+A	2_months	0,00E+00
culture_2	Insertion	*mkh1*	2517	1,2 %	A	+TACCTTACCATAAG	+CTTATGGTAAGGTA	2_months	2,19E-34
culture_2	SNP	*mkh1*	3145	0,2 %	C	A	G>T	2_months	1,06E-02
culture_2	Deletion	*pmk1*	251	1,7 %	T	-A	-A	2_months	2,26E-24
culture_2	SNP	*pmk1*	592	2,5 %	G	A	G>A	2_months	3,35E-100
culture_2	Deletion	*pmk1*	1097	10,4 %	A	-ACCCAACAGTCAACAT	-ACCCAACAGTCAACAT	2_months	2,58E-317
culture_2	Insertion	*pmc1*	2441	1,2 %	G	+CAGTAACAATATTATCAC	+GTGATAATATTGTTACTG	2_months	8,66E-12
**total = 64.7%**									
culture_2	Insertion	*sgf73*	896	43,5 %	T	+A	+T	3_months	0,00E+00
culture_2	Deletion	*win1*	2339	0,2 %	T	-ACGTA	-ACGTA	3_months	1,75E-04
culture_2	SNP	*sty1*	188	0,7 %	C	G	G>C	3_months	5,97E-27
culture_2	Insertion	*mkh1*	640	5,3 %	G	+T	+A	3_months	1,39E-199
culture_2	SNP	*pmk1*	592	2,7 %	G	A	G>A	3_months	2,86E-100
**total = 52,4%**									
culture_3	Insertion	*win1*	383	0,5 %	G	+ATACTAGTAAT	+ATACTAGTAAT	2_months	1,12E-15
culture_3	Insertion	*win1*	394	1,6 %	T	+CTACTACATC	+CTACTACATC	2_months	1,15E-77
culture_3	Deletion	*win1*	504	1,7 %	C	-A	-A	2_months	1,00E-162
culture_3	Deletion	*win1*	556	0,6 %	A	-G	-G	2_months	2,48E-19
culture_3	Insertion	*win1*	1273	0,6 %	A	+TATCCTTCACGTCGTTC	+TATCCTTCACGTCGTTC	2_months	8,84E-07
culture_3	SNP	*win1*	1859	0,6 %	C	T	C>T	2_months	2,15E-18
culture_3	Deletion	*win1*	2363	3,8 %	G	-TTGATTCCCCTAA	-TTGATTCCCCTAA	2_months	0,00E+00
culture_3	Deletion	*win1*	2545	0,7 %	C	-CGTT	-CGTT	2_months	3,27E-26
culture_3	Insertion	*win1*	2768	0,7 %	A	+ATCTAAAATTAAGATTAAAGCTGGAATTAAAGTCCAAATGAATTCAACAATTG	+ATCTAAAATTAAGATTAAAGCTGGAATTAAAGTCCAAATGAATTCAACAATTG	2_months	2,24E-25
culture_3	Deletion	*win1*	3680	3,5 %	C	-A	-A	2_months	1,28E-90
culture_3	SNP	*win1*	4179	0,2 %	G	T	G>T	2_months	1,29E-04
culture_3	Deletion	*mkh1*	236	1,2 %	A	-T	-A	2_months	3,22E-58
culture_3	Insertion	*mkh1*	640	15,8 %	G	+T	+A	2_months	0,00E+00
culture_3	Complex	*mkh1*	1884	0,2 %	G	A	CT>TG	2_months	4,53E-04
culture_3	Insertion	*mkh1*	2254	0,7 %	G	+A	+T	2_months	1,15E-03
culture_3	SNP	*mkh1*	3124	0,2 %	C	A	G>T	2_months	2,59E-02
culture_3	SNP	*mkh1*	3137	0,5 %	A	C	T>G	2_months	4,67E-16
culture_3	Deletion	*pmk1*	435	0,7 %	T	-TA	-TA	2_months	1,49E-13
culture_3	Insertion	*pmk1*	902	0,2 %	A	+GACGTATTTCGGTT	+GACGTATTTCGGTT	2_months	5,64E-03
**total = 34.0%**									
culture_3	Insertion	*win1*	383	0,2 %	G	+ATACTAGTAAT	+ATACTAGTAAT	3_months	1,40E-07
culture_3	Insertion	*win1*	394	1,1 %	T	+CTACTACATC	+CTACTACATC	3_months	2,14E-93
culture_3	Deletion	*win1*	504	1,1 %	C	-A	-A	3_months	1,14E-153
culture_3	Deletion	*win1*	556	0,7 %	A	-G	-G	3_months	3,91E-42
culture_3	Insertion	*win1*	1273	0,2 %	A	+TATCCTTCACGTCGTTC	+TATCCTTCACGTCGTTC	3_months	1,78E-02
culture_3	SNP	*win1*	1859	0,8 %	C	T	C>T	3_months	5,76E-57
culture_3	Deletion	*win1*	2363	3,8 %	G	-TTGATTCCCCTAA	-TTGATTCCCCTAA	3_months	0,00E+00
culture_3	Deletion	*win1*	2545	0,9 %	C	-CGTT	-CGTT	3_months	1,55E-60
culture_3	Insertion	*win1*	2768	0,6 %	A	+ATCTAAAATTAAGATTAAAGCTGGAATTAAAGTCCAAATGAATTCAACAATTG	+ATCTAAAATTAAGATTAAAGCTGGAATTAAAGTCCAAATGAATTCAACAATTG	3_months	4,17E-36
culture_3	Deletion	*win1*	3680	4,4 %	C	-A	-A	3_months	2,17E-146
culture_3	Deletion	*mkh1*	236	1,4 %	A	-T	-A	3_months	1,43E-105
culture_3	Insertion	*mkh1*	640	21,5 %	G	+T	+A	3_months	0,00E+00
culture_3	Insertion	*mkh1*	2593	0,2 %	C	+GGCCATTAATAGTTTGTGGTATTTCAACTTGCTTTACT	+AGTAAAGCAAGTTGAAATACCACAAACTATTAATGGCC	3_months	2,01E-03
culture_3	Deletion	*mkh1*	3340	1,2 %	C	-T	-A	3_months	6,81E-89
culture_3	Deletion	*pmk1*	435	0,3 %	T	-TA	-TA	3_months	4,45E-06
culture_3	SNP	*pmc1*	280	0,2 %	C	A	G>T	3_months	1,79E-03
**total = 38.6%**									
subculture_1	Complex	*win1*	357	1.2%	A	-TCGTGCT*T>A*T>A*C>A	-TCGTGCT*T>A*T>A*C>A	2_months	6,00E-213
subculture_1	Insertion	*win1*	394	3.4%	T	+CTACTACATCCTC	+CTACTACATCCTC	2_months	0,00E+00
subculture_1	Insertion	*win1*	397	0.2%	A	+CTACATCCTC	+CTACATCCTC	2_months	6,41E-13
subculture_1	Deletion	*win1*	506	0.2%	A	-AT	-AT	2_months	2,90E-18
subculture_1	SNP	*win1*	602	0.2%	C	G	C>G	2_months	3,77E-06
subculture_1	Insertion	*win1*	1273	0.3%	A	+TATCCTTCACGTCGTTC	+TATCCTTCACGTCGTTC	2_months	6,94E-04
subculture_1	SNP	*win1*	2478	1.8%	T	A	T>A	2_months	0,00E+00
subculture_1	Insertion	*win1*	3010	2.5%	T	+A	+A	2_months	0,00E+00
subculture_1	Deletion	*win1*	3305	0.5%	G	-AGAA	-AGAA	2_months	1,55E-72
subculture_1	Insertion	*win1*	3314	0.3%	C	+TA	+TA	2_months	1,62E-35
subculture_1	SNP	*win1*	3319	0.3%	A	G	A>G	2_months	4,13E-31
subculture_1	Insertion	*win1*	3544	0.2%	C	+GTATCGTATTATG	+GTATCGTATTATG	2_months	9,61E-13
subculture_1	Deletion	*win1*	3752	0.3%	C	-TT	-TT	2_months	5,54E-06
subculture_1	SNP	*win1*	4195	0.3%	G	T	G>T	2_months	2,85E-31
subculture_1	SNP	*wis1*	868	1.6%	C	G	G>C	2_months	0,00E+00
subculture_1	SNP	*wis1*	1108	3.4%	A	C	T>G	2_months	0,00E+00
subculture_1	SNP	*wis1*	1493	0.3%	T	C	A>G	2_months	4,84E-14
subculture_1	Insertion	*wis1*	1740	1.0%	T	+A	+T	2_months	1,94E-57
subculture_1	SNP	*sty1*	562	0.2%	A	G	T>C	2_months	5,44E-06
subculture_1	Insertion	*mkh1*	640	0.6%	G	+T	+A	2_months	2,15E-57
subculture_1	SNP	*tif452*	390	4.3%	G	T	G>T	2_months	0,00E+00
**total = 23.1%**									
subculture_2	Insertion	*win1*	394	2.4%	T	+CTACTACATCCTC	+CTACTACATCCTC	2_months	0,00E+00
subculture_2	SNP	*win1*	1100	1.0%	G	A	G>A	2_months	4,16E-206
subculture_2	Insertion	*win1*	1273	0.6%	A	+TATCCTTCACGTCGTTC	+TATCCTTCACGTCGTTC	2_months	2,96E-32
subculture_2	SNP	*win1*	2519	0.4%	C	A	C>A	2_months	1,50E-57
subculture_2	Deletion	*win1*	3489	1.1%	T	-C	-C	2_months	1,62E-131
subculture_2	Deletion	*win1*	3833	0.2%	G	-AACAGTT	-AACAGTT	2_months	2,25E-09
subculture_2	SNP	*wis1*	1380	4.6%	A	T	T>A	2_months	0,00E+00

subculture_2	SNP	*wis1*	1511	1.0%	A	C	T>G	2_months	5,00E-286
subculture_2	SNP	*sty1*	553	12.5%	T	A	A>T	2_months	0,00E+00
**total = 23.8%**									
subculture_3	Deletion	*win1*	292	0.6%	A	-CAGCCTC	-CAGCCTC	2_months	1,59E-79
subculture_3	Insertion	*win1*	394	0.4%	T	+CTACTACATCCTC	+CTACTACATCCTC	2_months	8,93E-42
subculture_3	Deletion	*win1*	1007	0.2%	C	-GATCT	-GATCT	2_months	1,32E-07
subculture_3	Insertion	*win1*	1273	1.8%	A	+TATCCTTCACGTCGTTC	+TATCCTTCACGTCGTTC	2_months	2,71E-84
subculture_3	Deletion	*win1*	1365	0.8%	A	-TG	-TG	2_months	1,12E-31
subculture_3	Deletion	*win1*	1846	0.8%	A	-T	-T	2_months	7,47E-185
subculture_3	Deletion	*win1*	1877	1.6%	A	-T	-T	2_months	0,00E+00
subculture_3	Insertion	*win1*	2179	1.8%	C	+TTCCGAAAATTGATGAG	+TTCCGAAAATTGATGAG	2_months	2,01E-31
subculture_3	Insertion	*win1*	3010	2.8%	T	+A	+A	2_months	0,00E+00
subculture_3	Insertion	*win1*	3105	0.7%	G	+T	+T	2_months	7,29E-41
subculture_3	Insertion	*win1*	3716	0.6%	G	+T	+T	2_months	2,92E-27
subculture_3	Insertion	*win1*	3880	0.6%	A	+T	+T	2_months	4,28E-24
subculture_3	SNP	*win1*	4177	0.5%	C	T	C>T	2_months	3,36E-91
subculture_3	SNP	*wis1*	944	9.2%	A	C	T>G	2_months	0,00E+00
subculture_3	Complex	*wis1*	1281	8.5%	GG	AA	AA	2_months	0,00E+00
subculture_3	SNP	*wis1*	1329	0.4%	T	A	A>T	2_months	1,91E-24
subculture_3	Insertion	*wis1*	1740	6.2%	T	+A	+T	2_months	0,00E+00
subculture_3	SNP	*sty1*	1	1.0%	T	C	A>G	2_months	5,77E-103
subculture_3	SNP	*sty1*	481	3.5%	C	A	G>T	2_months	0,00E+00
subculture_3	SNP	*sty1*	535	1.4%	A	G	T>C	2_months	0,00E+00
subculture_3	Insertion	*mkh1*	640	1.0%	G	+T	+A	2_months	1,76E-144
subculture_3	SNP	*mkh1*	1885	0.2%	A	G	T>C	2_months	8,16E-08
subculture_3	Deletion	*pmc1*	1296	0.1%	T	-TCTG	-CAGA	2_months	2,32E-02
**total = 43.4%**									
subculture_4	Deletion	*win1*	241	0.2%	C	-AAGGCGAGCGCCAAAGAGGACTTATTTTCAGAAGCTTTCAGAATGGCTG	-AAGGCGAGCGCCAAAGAGGACTTATTTTCAGAAGCTTTCAGAATGGCTG	2_months	8,39E-19
subculture_4	Insertion	*win1*	394	0.5%	T	+CTACTACATCCTC	+CTACTACATCCTC	2_months	4,48E-52
subculture_4	Deletion	*win1*	2982	2.4%	C	-AT	-AT	2_months	3,69E-312
subculture_4	Insertion	*win1*	3010	0.8%	T	+A	+A	2_months	3,73E-83
subculture_4	Deletion	*win1*	3045	1.2%	G	-A	-A	2_months	1,68E-129
subculture_4	Deletion	*win1*	3050	0.8%	T	-G	-G	2_months	1,17E-71
subculture_4	Deletion	*win1*	3097	0.2%	T	-GC	-GC	2_months	5,36E-04
subculture_4	Deletion	*win1*	3352	3.6%	G	-ATTCGA	-ATTCGA	2_months	0,00E+00
subculture_4	Deletion	*win1*	3374	0.6%	C	-TTATAGGTAGCGGTTCT	-TTATAGGTAGCGGTTCT	2_months	2,16E-77
subculture_4	Insertion	*win1*	3507	0.5%	A	+AATGTTAGTACTTGAACTCTTTGATCATCCT	+AATGTTAGTACTTGAACTCTTTGATCATCCT	2_months	5,87E-53
subculture_4	Deletion	*win1*	3617	0.5%	C	-TTTTCGAATTTCTACG	-TTTTCGAATTTCTACG	2_months	7,73E-52
subculture_4	Deletion	*win1*	3618	0.2%	T	-TTTCGAA	-TTTCGAA	2_months	1,13E-16
subculture_4	Deletion	*win1*	3682	0.8%	A	-T	-T	2_months	5,36E-49
subculture_4	Deletion	*win1*	3817	0.3%	T	-TC	-TC	2_months	1,06E-09
subculture_4	SNP	*wis1*	1588	1.9%	A	C	C	2_months	0,00E+00
subculture_4	Insertion	*wis1*	1740	1.2%	T	+A	+A	2_months	2,56E-167
subculture_4	SNP	*sty1*	35	1.0%	G	C	C	2_months	3,24E-122
subculture_4	Insertion	*mkh1*	640	1.6%	G	+T	+T	2_months	7,16E-247
subculture_4	Insertion	*mkh1*	2396	0.8%	G	+C	+C	2_months	2,87E-157
subculture_4	Insertion	*mkh1*	2846	0.2%	T	+TTAGA	+TTAGA	2_months	4,00E-02
subculture_4	SNP	*mkh1*	3152	0.2%	T	C	C	2_months	1,93E-06
**total = 19.5%**									
subculture_5	Deletion	*win1*	314	0.7%	A	-C	-C	2_months	5,12E-95
subculture_5	Deletion	*win1*	370	0.2%	C	-GTAA	-GTAA	2_months	2,78E-08
subculture_5	Insertion	*win1*	394	4.0%	T	+CTACTACATCCTC	+CTACTACATCCTC	2_months	0,00E+00
subculture_5	Deletion	*win1*	942	0.7%	C	-A	-A	2_months	6,42E-94
subculture_5	Deletion	*win1*	1024	2.0%	G	-C	-C	2_months	0,00E+00
subculture_5	SNP	*win1*	1129	0.4%	A	T	A>T	2_months	1,33E-45
subculture_5	SNP	*win1*	1251	0.2%	A	G	A>G	2_months	1,47E-02
subculture_5	Deletion	*win1*	1258	0.9%	T	-A	-A	2_months	3,32E-54
subculture_5	Deletion	*win1*	1265	2.4%	G	-AACGT	-AACGT	2_months	2,37E-189
subculture_5	Insertion	*win1*	1273	0.8%	A	+TATCCTTCACGTCGTTC	+TATCCTTCACGTCGTTC	2_months	1,43E-37
subculture_5	Deletion	*win1*	2376	0.2%	A	-TATACT	-TATACT	2_months	8,56E-05
subculture_5	Insertion	*win1*	3010	1.8%	T	+A	+A	2_months	0,00E+00
subculture_5	Deletion	*win1*	3142	0.2%	C	-CTTGTTCGAAATAATTCCGACCGAAGTCGTTTAT	-CTTGTTCGAAATAATTCCGACCGAAGTCGTTTAT	2_months	3,77E-03
subculture_5	Deletion	*win1*	3407	0.5%	C	-GT	-GT	2_months	6,46E-59
subculture_5	Deletion	*win1*	3626	0.3%	T	-TTCTACGTTAC	-TTCTACGTTAC	2_months	4,45E-26
subculture_5	Insertion	*win1*	3922	0.3%	A	+GGAACACCTACGTATAT	+GGAACACCTACGTATAT	2_months	1,44E-09
subculture_5	SNP	*win1*	4177	0.9%	C	T	C>T	2_months	1,02E-204
subculture_5	SNP	*wis1*	1088	1.0%	A	C	T>G	2_months	5,31E-116
subculture_5	SNP	*wis1*	1182	5.2%	A	C	T>G	2_months	0,00E+00
subculture_5	SNP	*wis1*	1427	0.7%	C	G	G>C	2_months	5,14E-50
subculture_5	SNP	*wis1*	1430	0.2%	C	T	G>A	2_months	2,63E-02
subculture_5	SNP	*sty1*	545	13.2%	G	A	C>T	2_months	0,00E+00
subculture_5	SNP	*sty1*	558	1.4%	T	G	A>C	2_months	0,00E+00
subculture_5	SNP	*sty1*	646	0.7%	C	T	G>A	2_months	9,02E-130
subculture_5	SNP	*sty1*	659	10.2%	A	T	T>A	2_months	0,00E+00
subculture_5	Insertion	*mkh1*	640	1.6%	G	+T	+A	2_months	0,00E+00
subculture_5	Deletion	mkh1	3302	0.2%	G	-TTGAATTCT	-AGAATTCAA	2_months	2,16E-06
**total = 50.9%**									
subculture_6	Deletion	*win1*	292	0.8%	A	-CAGCCTC	-CAGCCTC	2_months	8,24E-121
subculture_6	Insertion	*win1*	394	4.5%	T	+CTACTACATCCTC	+CTACTACATCCTC	2_months	0,00E+00
subculture_6	Insertion	*win1*	397	1.4%	A	+CTACATCCTC	+CTACATCCTC	2_months	5,06E-294
subculture_6	Deletion	*win1*	666	0.6%	G	-A	-A	2_months	7,66E-43
subculture_6	Deletion	*win1*	972	0.7%	A	-TATGTTGACTTCAGTTC	-TATGTTGACTTCAGTTC	2_months	8,61E-99
subculture_6	Deletion	*win1*	1253	0.5%	C	-TTGA	-TTGA	2_months	8,32E-13
subculture_6	Insertion	*win1*	1273	0.4%	A	+TATCCTTCACGTCGTTC	+TATCCTTCACGTCGTTC	2_months	5,44E-06
subculture_6	Deletion	*win1*	1644	0.6%	A	-T	-T	2_months	5,80E-08
subculture_6	Deletion	*win1*	1681	0.8%	C	-A	-A	2_months	6,91E-152
subculture_6	SNP	*win1*	1749	0.9%	C	T	C>T	2_months	5,12E-184
subculture_6	Deletion	*win1*	1905	1.0%	T	-C	-C	2_months	8,92E-195
subculture_6	Insertion	*win1*	2313	4.8%	C	+G	+G	2_months	0,00E+00
subculture_6	Insertion	*win1*	2512	0.7%	G	+GA	+GA	2_months	1,57E-68
subculture_6	SNP	*win1*	2762	2.2%	C	T	C>T	2_months	0,00E+00
subculture_6	Insertion	*win1*	3010	1.1%	T	+A	+A	2_months	2,47E-254
subculture_6	Insertion	*win1*	3105	1.2%	G	+TA	+TA	2_months	4,04E-96
subculture_6	Deletion	*win1*	3401	1.2%	G	-T	-T	2_months	4,04E-182
subculture_6	Insertion	*win1*	3507	1.2%	A	+AATGTTAGTACTTGAACTCTTTGATCATCCT	+AATGTTAGTACTTGAACTCTTTGATCATCCT	2_months	1,95E-173
subculture_6	SNP	*win1*	3546	1.2%	C	A	C>A	2_months	7,68E-165
subculture_6	Deletion	*win1*	3551	1.1%	T	-A	-A	2_months	5,04E-156
subculture_6	Deletion	*win1*	3682	1.3%	A	-T	-T	2_months	4,67E-78
subculture_6	Deletion	*win1*	3783	0.4%	C	-AGAT	-AGAT	2_months	8,43E-12
subculture_6	Complex	*win1*	3784	1.0%	T	T>C*-C	-T>C*-C	2_months	1,50E-50
subculture_6	Deletion	*win1*	3816	1.3%	C	-T	-T	2_months	4,48E-71
subculture_6	SNP	*win1*	4177	0.4%	C	T	C>T	2_months	1,27E-48
subculture_6	Insertion	*wis1*	1740	1.2%	T	+A	+T	2_months	4,31E-108
subculture_6	SNP	*sty1*	535	4.8%	A	G	T>C	2_months	0,00E+00
subculture_6	Insertion	*mkh1*	640	2.2%	G	+T	+A	2_months	0,00E+00
subculture_6	SNP	*tif452*	260	1.0%	T	C	T>C	2_months	1,75E-295
subculture_6	SNP	*tif452*	383	0.5%	C	G	C>G	2_months	5,97E-28
**total = 41.0%**									
**total number of mutations from independent cultures and subcultures = 195**									

Supplementary Table 5. Mutations identified in the targeted resequencing experiment. The actual presence of some mutations in the population at two and three months was confirmed by Sanger sequencing (red alleles): one hundred and twenty cells per frozen pool from the two and three-month-old cultures were plated out and scored for their sensitivity to temperature, calcium, HU and SDS. The DNA of the sensitive colonies was prepared and used to amplify and sequence by Sanger the relevant target genes.

Supplementary Table 6: No increase in cell number (cells/ml) is observed after addition of traces of glutamate, ammonium chloride or yeast extract to 3-day-old cultures of the indicated strains.

Strains	day 3	day 6			
		Glu	NH_4Cl	YE	Mock
wild-type	3.8E+06	4.6E+06	4.4E+06	4.6E+06	4.0E+06
sgf73-896 +T	4.0E+06	4.7E+06	4.3E+06	4.4E+06	4.0E+06
pmk1-153 +A	3.9E+06	4.0E+06	4.0E+06	4.0E+06	3.8E+06
sty1-G197T	4.1E+06	4.4E+06	4.0E+06	4.2E+06	4.3E+06

Supplementary Table 6. Determination of an increase in cell number following addition of traces of nitrogen. No increase in cell number (cells/mL) is observed after addition of traces of glutamate, ammonium chloride or yeast extract to three-day-old cultures of the indicated strains. The number of cells was determined with a Beckman Coulter Z1 Particle Counter readout of a 1/100 dilution.

Supplementary Table 7. Primers Used in this Study

Primer name	Gene targeted	Primer Sequence 5' > 3'
OL1451	*sgf73*	AAAGTCGAAAATGCGTGG
OL1452	*sgf73*	TGTTTTGCTTGCAGCTTGT
OL1453	*sgf73*	TTCTTTTGCAGTTTCGAGG
OL1454	*sgf73*	TAAATCGACAGCTAAGGG
OL1455	*sgf73*	TTCTTTTCATGATTCCCAG
OL1456	*sgf73*	AAACTCGGTTCTTATCAA
OL1073	*win1*	TAGTTGCATTGACTTCGCT
OL1074	*win1*	TGGCCTTCTGTGATTTTCTT
OL1075	*win1*	CATCGTCGCTCTTTGACT
OL1076	*win1*	CTGTGCATATAATCGTCTTCCT
OL1077	*win1*	CTTCTTCCTCTTCCATTCC
OL1078	*win1*	CGAGTCCTCCTCATCATAA
OL1079	*win1*	CTTCTCTAAGCATTCGTC
OL1080	*win1*	TAGCACATCAACCATACC
OL1081	*win1*	GTGGATGGAAATATGGTGT
OL1082	*win1*	TGGTCCGTTTGAAATTTGAG
OL1083	*win1*	GGTTGGTAATGAGGAAATGG
OL1084	*win1*	TTGTTGAATGATATGGACGG
OL1085	*win1*	CGTCTTCTTTCTTTTCCGT
OL1086	*win1*	ATATTTCACCAACCAGCG
OL1087	*win1*	CCACATCGAACACGGATT
OL1088	*win1*	CGCATATGGAGGTACACTTT
OL1089	*win1*	CGAACATGAAGGAGCATAC
OL1090	*win1*	CACATCAAGCAATGACAAAG
OL1091	*win1*	CTCACTTGTTGATTCTGC
OL1092	*win1*	CCACACATTTTCCAACCT
OL1093	*win1*	CAGTTGGGTTGGATTCAT
OL1094	*win1*	CCTGTTCTCCTTTGTCGT
OL1095	*win1*	AAAGTAATAGAAGGGACCGA
OL1096	*win1*	CCTCAATTCTACCGTAAC
OL1097	*win1*	GAAGTGCATCGTGAAAAAG
OL1098	*win1*	AATATCCATAGCACCAACC
OL1099	*win1*	GCAGCATCTAGACCAAAA
OL1100	*win1*	TTCGACTACGGATGAGTT
OL1101	*win1*	CGTTGTTTTGTTTCTGATCCC
OL1102	*win1*	TCTCTTCCTAGCGCCGTA
OL1509	*wis1*	AACAGAGCAGGCAATTAGA
OL1510	*wis1*	ACATGGCTCCTGAAAGAA

OL1511	*wis1*	GATAAGCTCCTAAAGCCA
OL1512	*wis1*	TGGTGCCTTTTTTGTGGA
OL1513	*wis1*	ACTCCTTCGTCTTTGATACC
OL1514	*wis1*	CTAAACATTCCTCCCACCC
OL1515	*wis1*	GAAAATGTGGAAAAAGGGG
OL1516	*wis1*	ACAGGAGAAATTGAAGGC
OL1517	*wis1*	TCCGGCATTGATTTTGATCT
OL1518	*wis1*	CTTCTCCAAATAATCAACCC
OL1041	*sty1*	CATAACATACCCCGAGAACA
OL1042	*sty1*	CCGAGACCACGTTAACCA
OL1043	*sty1*	ATTACTTCCATAGGCGGC
OL1044	*sty1*	ACTACTTACATCGCGACC
OL1045	*sty1*	TGGGTTTCAGATCACGGT
OL1046	*sty1*	CCTTTGGCCTCGTTTGTT
OL1047	*sty1*	AACAGCGACATTCATTCCA
OL1048	*sty1*	ACATACCTCCTTACCACAAC
OL1329	*mkh1*	CGAGGAAACGCTAAGTAA
OL1330	*mkh1*	GCAAACCTGTCCATGCAA
OL1331	*mkh1*	TTCCAACACTACACATCC
OL1332	*mkh1*	TAGTGCAATATCTGGGTTTC
OL1333	*mkh1*	AATTCCGCAAACATCGAC
OL1334	*mkh1*	AAATACGACATAACGCGAG
OL1335	*mkh1*	TACAATCCTAGAGCCCCAAA
OL1336	*mkh1*	TCGCCTAAAACTCCTGAA
OL1337	*mkh1*	CCCAGTTACCAGAATTGA
OL1338	*mkh1*	CCGGACATAACGAAGCTA
OL1339	*mkh1*	GCTTCCACACTTTCCTTT
OL1340	*mkh1*	GACGTTTTCGAGGGTTTG
OL1341	*mkh1*	GCCATTTCTCCTTTAGCAC
OL1342	*mkh1*	GGCGGGGCTCAATACATA
OL1343	*mkh1*	CACTATCCATACCCGATTCT
OL1344	*mkh1*	CAAATTCGAACCCATTCCA
OL1345	*mkh1*	TACCCACTCACCTTGATCC
OL1346	*mkh1*	CTCTGGTTTCCGCTCTTC
OL1347	*mkh1*	CTTTTGAGGGACGAATTTTG
OL1348	*mkh1*	GTTCTGGGTATATGGCTG
OL1049	*pek1*	GGTTGTTTGTTTGCGAGG
OL1050	*pek1*	TATCCCGGCTCTCCTTTC
OL1051	*pek1*	TGGTGTTTTCTAGCGAGT
OL1052	*pek1*	TTTGTATATCGCGTCGAG
OL1053	*pek1*	GGAATATTGCGGAGCAGG

OL1054	*pek1*	AGGTAAAAGGGGAGGTGG
OL1055	*pek1*	GGTTTAACATTGATGGAGG
OL1056	*pek1*	AACAAGCGAACTGGAAGA
OL1057	*pek1*	AGAGTTCCTTCGTCAAGT
OL1058	*pek1*	AAGCTAGCAAGGCGTAAA
OL1059	*pmk1*	TTTTTCCACATCCCTTTTGCC
OL1060	*pmk1*	AATCAATCACAGGAAGCCG
OL1061	*pmk1*	GAGAGAGATAAAACTGCTGA
OL1062	*pmk1*	TCGCGTAACATACCAACT
OL1063	*pmk1*	TGCATTTACGATCTCGAC
OL1064	*pmk1*	TGATAGCTGCTGAACGAA
OL1065	*pmk1*	TGGTTTTATGACGGAGTATG
OL1066	*pmk1*	GAAATACGTCTGTTGGGG
OL1067	*pmk1*	ACAGGAGTATGTTCGAAG
OL1068	*pmk1*	AGGTTTGAGAGGATGAAG
OL1069	*pmk1*	CTCATCCAACAAACCCAAC
OL1070	*pmk1*	GCAAGAAAAGGGAGCACA
OL1201	*pmk1*	CTTGCATTTACGATCTCGAC
OL1202	*pmk1*	ACACTCCAAACATCAATACC
OL1173	*tif452*	AACCCAATTACGACCACC
OL1174	*tif452*	CCCCAAAACTCTTCCACT
OL1175	*tif452*	AAAAACACACCCCCTTCA
OL1176	*tif452*	CACCAGTAACTTCTTTCCCA
OL1177	*tif452*	ACTGCTGTGATATCGACT
OL1178	*tif452*	TCGTAAATGAACACCCCT
OL1293	*pmc1*	GGCGATTTCCAACGAGAA
OL1294	*pmc1*	GAACCCACATCTCCCAAC
OL1295	*pmc1*	GAGTTGTTGAGTAGGTGG
OL1296	*pmc1*	CGGAGATTGGATAACAAG
OL1297	*pmc1*	GCACCTCCGAAAAAAACAA
OL1298	*pmc1*	TCGACTGACCAAAGCTCT
OL1299	*pmc1*	TCTCAGGTTTCCTCTTTAACAC
OL1300	*pmc1*	TTGCACGCTCATCTCCTT
OL1301	*pmc1*	CTTTTTTTAGTGCAGGCG
OL1302	*pmc1*	TCCCAAAGATATTCCCACCA
OL1303	*pmc1*	GTCAAACACACGATAGCAT
OL1304	*pmc1*	ACTTCGGGACTTAACTCT
OL1305	*pmc1*	CCTTGGGGTGTCAGAATTA
OL1306	*pmc1*	AGAACAAGGGTCAAGAGT
OL1307	*pmc1*	AGCAAAAGCCAAAGCCAA
OL1308	*pmc1*	GAAAGAACATCGCCGGAC

OL1309	*pmc1*	AATTAACACCGACAGCAG
OL1310	*pmc1*	GTGTCGAATGGGTGGAAG
OL1311	*pmc1*	CAGTCATTCACACCACCCA
OL1312	*pmc1*	ATTTCTACTCACGAACCTCA
OL1313	*pmc1*	CTCATCTGCAATTTATCGGC
OL1314	*pmc1*	TTCCTCGGTGTTCCTTCT
OL1315	*pmc1*	CCACTTCGAGAGCTATTT
OL1316	*pmc1*	TCTCCTCTTCATCTTCTCTT

SUPPLEMENTARY METHODS

Whole Genome and Targeted Sequencing Analysis

Library construction was performed using Illumina TrueSeq DNA PCR-Free Prep Kit following the manufacturer's instructions. Paired end sequencing was achieved with either Illumina HiSeq 2500 (250 nucleotide-long reads at Novogene Bioinformatics Technology Company LTD) or an Illumina MiSeq System (300 nucleotide-long reads at the Pasteur Institute) technology following the manufacturer's instructions. The preprocessing step was performed with fqCleanER (v.5.01) which includes the trimming of short and aberrant reads with AlienTrimmer [2], correcting sequencing mistakes with Musket [3], and merging the overlapping reads using Flash [4]. The remaining reads were aligned to the *S. pombe* ASM294v2.23 (strain 972 h^-) reference genome with Burrows-Wheeler Aligner (v.0.7.5a.) [5]. The resulting reads were sorted and indexed with Samtools (v.0.1.19) and duplicated reads were marked with Picard tools (v.1.96). Indel realignment, removing the duplicates, variant filtering and calling was performed according to the GATK best practices using GATK version 2.7-2 [6]. The output .vcf file was annotated using snpEff (v.3.5) [1]. In the targeted resequencing experiments, the aligned reads were sorted and indexed, further processed with Samtools (v.1.3) and converted to the .mpileup format. We used VarScan [7] to call the variants. According to the experimental design that we have developed, we only consider variants from the positions covered at least 5,000 times exhibiting an allele frequency above 0.1%. To distinguish mutations from sequencing errors, we have applied several filters. First, based on the comparison of the distribution of the reference and alternative variants on the

reads, VarScan calculates a p-value based on the Fisher's exact test. Second, we discarded the variants with a base quality below 35 and exhibiting more than a 7% difference in quality with the reference allele. Third, we retained the alleles for which mutations are present in equimolar proportions on the paired reads (within a 20% range). In addition, in the six-subculture experiment in which two independent PCR reactions were performed and sequenced, we have considered as true positive variants only those that were found in both batches. Using this approach, we are able to detect both SNVs and indels present at a 0.1% frequency, and short indels (± 1 nucleotides) at frequency of 0.5%.

REFERENCES

1. Cingolani, P. *et al.* A program for annotating and predicting the effects of single nucleotide polymorphisms, SnpEff. *Fly* **6,** 80–92 (2014).
2. Criscuolo, A. & Brisse, S. AlienTrimmer: A tool to quickly and accurately trim off multiple short contaminant sequences from high-throughput sequencing reads. *Genomics* **102,** 500–506 (2013).
3. Liu, Y., Schröder, J. & Schmidt, B. Musket: a multistage k-mer spectrum-based error corrector for Illumina sequence data. *Bioinformatics* **29,** 308–315 (2012).
4. Magoc, T. & Salzberg, S. L. FLASH: fast length adjustment of short reads to improve genome assemblies. *Bioinformatics* **27,** 2957–2963 (2011).
5. Li, H. & Durbin, R. Fast and accurate short read alignment with Burrows-Wheeler transform. *Bioinformatics* **25,** 1754–1760 (2009).
6. McKenna, A. *et al.* The Genome Analysis Toolkit: A MapReduce framework for analyzing next-generation DNA sequencing data. *Genome Res.* **20,** 1297–1303 (2010).

7. Koboldt, D. C. *et al.* VarScan: variant detection in massively parallel sequencing of individual and pooled samples. *Bioinformatics* **25,** 2283–2285 (2009).
